# Single-cell and spatial transcriptomics reveal P4HA2-mediated radiotherapy resistance mechanisms in breast cancer

**DOI:** 10.7150/thno.121257

**Published:** 2026-01-01

**Authors:** Huimin Li, Junzhi Liu, Yuheng Jiao, Fengyu Xu, Shurui Wang, Qiang Tang

**Affiliations:** 1 The Second Affiliated Hospital, School of Medicine, Zhejiang University, Hangzhou, Zhejiang, 310009, China.; 2 Department of Oncology, Shanghai Pulmonary Hospital, School of Medicine, Tongji University, Shanghai, 200433, China.; 3 Department of Heart failure, Shanghai East Hospital, School of Medicine, Tongji University, Shanghai, 200120, China.; 4 Chinese Academy of Medical Sciences & Peking Union Medical College, Beijing 100730, China.

**Keywords:** radiotherapy resistance, scRNA, breast cancer, *P4HA2*, machine learning

## Abstract

**Background:** Radiotherapy resistance in breast cancer remains a major clinical challenge. The key molecular determinants and cellular populations driving this resistance are not fully understood.

**Methods:** A radiotherapy resistance (RR) gene panel was identified from TCGA-BRCA and GSE120798 cohorts. Single-cell and spatial transcriptomics characterized RRhigh epithelial cells (RRhighepi). A prognostic model, named SuperPC and StepCox-based Radiotherapy Resistance model (SSRR), was built via machine learning and Mendelian randomization. Functional roles of Prolyl 4-Hydroxylase Subunit Alpha 2 (*P4HA2*) were validated *in vitro*.

**Results:** The RR gene panel was upregulated in tumors and enriched for cell cycle pathways. RRhighepi cells exhibited elevated stemness, activated cell cycle and metabolic programs, and enhanced DNA damage repair. RRhighepi represented a developmental origin and communicated with endothelial cells. The SSRR model stratified patients into high-risk groups with poorer survival and distinct therapeutic responses. *P4HA2*, a key model gene, was upregulated in multiple cancers. *P4HA2* knockdown suppressed proliferation, invasion, and colony formation, and synergized with radiotherapy to reduce stemness and enhance DNA damage. WGCNA confirmed co-module membership of *P4HA2* and the RR panel.

**Conclusions:** This study, through multi-omics analysis, proposes a potential mechanistic model associated with radiotherapy resistance in breast cancer. *P4HA2* is a potential therapeutic target that sensitizes breast cancer to radiotherapy. The RR gene panel and SSRR model provide insights into resistance mechanisms and prognostic stratification.

## Introduction

Radiotherapy resistance in breast cancer remains a critical concern in clinical oncology, both in terms of incidence and evolving epidemiological trends. A population-based study utilizing the Surveillance, Epidemiology, and End Results (SEER) database analyzed 374,993 patients, of whom 154,697 received radiotherapy. With a median follow-up of 8.9 years, 13% of patients developed second primary malignancies. The incidence of secondary cancers was significantly higher in those who underwent radiotherapy, particularly among younger individuals and those with longer latency periods 1. Variations in reported incidence across studies may be attributable to differences in patient cohorts, radiotherapy protocols, and evaluation criteria. A separate investigation involving 1,003 breast cancer patients revealed subtype-specific differences in radiotherapy response following breast-conserving surgery. Human epidermal growth factor receptor 2-positive (*HER2+*) tumors exhibited the highest level of radioresistance, whereas patients with triple-negative breast cancer (TNBC) derived the greatest reduction in breast cancer-specific mortality from radiotherapy 2.

Aberrations in cell cycle regulation are closely associated with clinical outcomes in breast cancer. Numerous studies have demonstrated that dysregulation of cell cycle-associated proteins and their encoding genes significantly influences disease progression and patient survival. For example, overexpression of *cyclin D1* mRNA is strongly correlated with poor prognosis in estrogen receptor-positive (*ER+*) breast cancer. In a study of 253 primary breast cancer cases, elevated cyclin D1 mRNA levels in *ER+* tumors were significantly associated with increased risk of recurrence (P = 0.0016), local relapse (P = 0.025), distant metastasis (P = 0.019), and mortality (P = 0.025), whereas no such association was observed in *ER*-negative tumors 3. Similarly, low expression of F-box and WD repeat domain containing 7 (*FBXW7*), a gene involved in cell cycle regulation, correlates with adverse prognosis. Specifically, *FBXW7* mRNA levels were markedly reduced in high-grade tumors and hormone receptor-negative subtypes, with lower expression predicting poorer breast cancer-specific survival 4. Elevated *Cullin 7* expression has also been associated with advanced pathological stage (P = 0.013), lymph node metastasis (P = 0.022), and decreased overall survival (P = 0.037). Knockdown of *Cullin 7* inhibited breast cancer cell proliferation and invasion, likely through modulation of cell cycle-associated proteins 5. Collectively, these findings underscore the prognostic significance of cell cycle dysregulation and highlight related molecules as candidate biomarkers and therapeutic targets.

Metabolic reprogramming plays a pivotal role in radiotherapy resistance in breast cancer. Hallmark metabolic alterations, such as the Warburg effect and enhanced lipid biosynthesis, are linked to chemotherapy failure, and the distinct metabolic profiles of metastatic lesions contribute to resistance to both targeted and immune therapies 6. Multiple mechanisms underlie this phenomenon. For instance, Pyruvate dehydrogenase kinase 1 (*PDK1*)-dependent metabolic reprogramming has been shown to drive metastatic potential. Liver metastases of breast cancer display unique metabolic adaptations characterized by elevated *PDK1* expression, which is essential for metabolic fitness and hepatic colonization 7. In TNBC, Retinoic acid receptor responder 2 (*RARRES2*) mediates brain metastasis via lipid metabolism reprogramming. Downregulation of *RARRES2* is associated with brain tropism and modulates the *PTEN-mTOR-SREBP1* axis to alter glycerophospholipid and triglyceride levels, thereby promoting tumor cell survival in the brain microenvironment 8.

The tumor microenvironment (TME) also exerts critical influence on radiotherapy resistance in breast cancer. Radiotherapy induces profound alterations in the vascular, stromal, and immune components of the TME, potentially facilitating tumor recurrence and resistance 9. Specific TME constituents, including cancer-associated fibroblasts (CAFs), tumor-associated macrophages (TAMs), and myeloid-derived suppressor cells (MDSCs), modulate immune evasion and contribute to therapeutic resistance 10. Fibroblast Growth Factor 2 (*FGF2*), a secreted factor within the TME, confers resistance to various therapies in *ER+* breast cancer. Mechanistically, *FGF2* activates *ERK1/2* signaling via *FGFRs*, leading to upregulation of *Cyclin D1* and downregulation of Bim. Inhibition of *FGF2* or its receptors reverses therapeutic resistance, and transcriptional signatures of *FGF2* signaling can predict relapse-free survival 11.

In this study, we integrated bulk RNA sequencing data from TCGA-BRCA and GSE120798 to identify a gene panel associated with radiotherapy resistance in breast cancer. Through differential expression analysis and downstream validation using single-cell and spatial transcriptomics, we delineated the expression patterns and potential functional roles of these genes within malignant epithelial cells. Particular attention was paid to the role of tumor epithelial cells in cell cycle regulation, metabolic reprogramming, and intercellular communication with endothelial cells, elucidating their central function in mediating radiotherapy resistance.

Additionally, we identified *P4HA2* as a potential therapeutic target. *P4HA2* is highly expressed in breast cancer and strongly correlates with poor prognosis. Its knockdown significantly suppressed proliferation and migration of breast cancer cells, suggesting a key role in the development of radioresistance. Taken together, this study presents a comprehensive multi-omics framework for decoding the regulatory landscape underlying breast cancer radioresistance and provides a theoretical foundation and candidate targets for future therapeutic interventions.

## Methods and Materials

### Cell Culture and Transfection Conditions

The HCC1806 and MCF7 cell lines were sourced from the Cell Bank of the Chinese Academy of Sciences (Shanghai, China) and were maintained in DMEM medium (Boster, China) supplemented with 10% FBS (HyClone, USA), incubated at 37 °C in a 5% CO_2_ atmosphere. To suppress *P4HA2* expression, siRNAs were transfected into these cells using Lipofectamine 3000. The siRNAs (including the scrambled negative control), used in this study were commercially acquired from Shanghai GenePharma Co., Ltd.

### Cell Viability Assay

Cells were digested, centrifuged, and seeded into 96-well plates at a density of 2000-3000 cells per well. Cell viability was measured using the Cell Counting Kit-8 (APExBIO, United States) at 0, 24, 48, and 72 h after treatment.

### Western Blotting (WB)

The cells were lysed in a cold buffer supplemented with phosphatase and protease inhibitors. Protein levels were quantified using the bicinchoninic acid assay. Following separation on 4-12% SDS/PAGE gels, the proteins were transferred onto PVDF membranes, blocked, and then incubated with both primary and secondary antibodies. Immunoreactive proteins were detected using a chemiluminescent solution. Detailed information on the antibodies used is provided in Table S1.

### Transwell Assays

Cell migration was evaluated using the Boyden chamber assay, employing an 8-μm pore size. A total of 1 × 10^5^ cells were suspended in 200 μL of medium lacking FBS and added to the upper chamber, while the lower chamber contained 20% FBS medium. After 24 h, cells were fixed and stained, and the number of cells in six randomly chosen fields was counted.

### EdU Assay

Cells were transfected and seeded in 24-well plates at a density of 5 × 10^4^ cells per well, followed by overnight incubation. To evaluate EdU incorporation, the EdU Cell Proliferation Kit, labeled with Alexa Fluor 488, was employed. EdU-positive cells were stained using Azide 488 and Hoechst 33342. Images were captured from three randomly chosen fields, and the EdU incorporation rate was calculated as: EdU-positive rate = (number of EdU-positive cells / (number of EdU-positive cells + number of EdU-negative cells)) × 100%.

### Colony Formation Assay

Cells (8 × 10^2^ to 1 × 10^3^ cells) were seeded into 6-well plates and incubated at 37 °C for 10 to 14 days. After incubation, colonies were fixed with methanol, stained with 1% crystal violet for 15 min, and counted to assess colony formation ability.

### Immunofluorescence Staining

Cells were seeded on 18 mm coverslips and stabilized overnight at 37 °C. Briefly, the slides were blocked with 5% bovine serum albumin, followed by incubation with primary antibodies and fluorescently labeled secondary antibodies. Nuclei were stained using DAPI (Thermo). The samples were visualized using an LSM 880 laser scanning microscope (Zeiss). Detailed information on the antibodies used is provided in Table S1.

### 3D Spheroid Assay

Cells are dissociated with 0.05% trypsin and quenched with complete medium followed by centrifugation. The cells were washed twice with PBS and centrifuged to remove serum before counting. A total of 5,000 cells are seeded per well in ultra-low attachment 6-well plates with 2 mL of DMEM/F12 specialized medium for sphere formation. After 2-3 days of culture, 1 mL of fresh medium is supplemented per well. Sphere growth and size are recorded after 7-10 days of culture.

### Data Sources

Single-cell RNA sequencing data GSE176078 and spatial transcriptomics data GSM6760695, GSM6760696, GSM6760697, as well as bulk transcriptomics data GSE16446, GSE20486, GSE24450, and GSE21656 were downloaded from the GEO database. Pan-cancer (including TCGA, ICGC) transcriptomics, genomics, methylation, and clinical data were sourced from the UCSC Xena database 12, GEO database, ArrayExpress database, and ICGC Data Portal. Immunohistochemical staining data were obtained from The Human Protein Atlas 13. Pan-cancer single-cell data were downloaded from the TISCH database 14, 15.

### Single-cell and Spatial Transcriptomics Processing

Single-cell RNA sequencing (scRNA-seq) data were processed using the Seurat package (v4.1.3) in R (v4.2.2) 16. Quality control measures included: 1) exclusion of genes present in fewer than three cells; 2) removal of cells expressing fewer than 50 total genes; and 3) elimination of cells with more than 5% mitochondrial gene expression. Data normalization was performed using the SCTransform method, while batch effects were corrected using the Harmony method (v0.1.0) to combine Seurat objects into a unified dataset. Dimensionality reduction was conducted via Principal Component Analysis (PCA), and cell classification was executed using the FindNeighbors and FindClusters functions. Cell-cycle scores were computed with Seurat's CellCycleScoring function. For visualization, the Uniform Manifold Approximation and Projection (UMAP) algorithm was applied. Cell types were identified through differential expression analysis with Seurat's FindAllMarkers function. The thresholds applied for marker gene identification were adjusted P-value < 0.05, expression > 0.25, and absolute log2 fold change > 0.5. SingleR was used for annotation based on marker gene composition, and validation was done using the CellMarker database. For cell communication analysis in single-cell transcriptomics, the Cellcall algorithm 17 was used to explore tumor microenvironment interactions in the RR group. Pseudotime analysis was conducted using the monocle2 algorithm 18-20 to identify developmental trajectories in the RR group. The CytoTRACE algorithm 21 was used to assess stemness features in the RR group, and transcription factor identification in single-cell data was performed using the SCENIC algorithm 22. Metabolic pathway activity was assessed using the scFEA algorithm 23 to determine metabolic differences in the RR group.

ST data were processed and visualized using Seurat. The data were standardized using the SCT method, and integration was performed using SelectIntegrationFeatures, PrepSCTIntegration, FindIntegrationAnchors, and IntegrateData functions. Unsupervised clustering methods were applied to group similar ST regions. Cell group annotations were based on hematoxylin and eosin (HE) stained sections and significantly variable genes within each group. Spatial transcript and feature plots (SpatialDimPlot and SpatialFeaturePlot) were used for data visualization. For cell-type decomposition of spatial transcriptomics data, Robust Cell Type Decomposition (RCTD) was used to align reference scRNA-seq cell types with spatial transcriptomics data. Cell type marker genes were identified using Seurat's FindAllMarkers function, with positive log2 fold change as the selection criterion. The standard RCTD analysis pipeline was applied, concentrating on reference data and Visium spatial transcriptomics data, with the dual-mode configured to full. Trajectory evolution in spatial transcriptomics was analyzed using the stLearn algorithm 24 to identify developmental trajectory differences in the RR group. MISTy 25 in mistyR (v1.2.1) was utilized to evaluate how the abundance of primary cell types influences the prevalence of other major cell types.

### Enrichment Analysis

The Metascape online analysis tool 26 was used to perform enrichment analysis of radiation resistance gene panels. Multiple gene lists were uploaded, and the top 20 pathways were selected for further analysis. Enrichment analysis for single-cell and spatial transcriptomics was performed using the irGSEA package 27. Gene sets were downloaded from the Molecular Signatures Database 28-30.

### Survival Analysis

To investigate risk models and the role of *P4HA2* in clinical diagnosis and prognosis, survival analysis was performed on pan-cancer transcriptomic cohorts, including BRCA, using the Survival (v3.2-10) and Survminer (v0.4.9) packages. The median value was used to divide patients into high or low categories, and Kaplan-Meier survival curves were generated using the survfit function.

### Immunotherapy Efficacy and Drug Sensitivity Prediction

Cohorts for immunotherapy analysis were sourced from the TIGER database 53. Drug sensitivity data for cell lines were obtained from the Cancer Therapeutics Response Portal (CTRP v.2.0, released October 2015, https://portals.broadinstitute.org/ctrp) and PRISM Repurposing dataset (19Q4, released December 2019, https://depmap.org/portal/prism/).

### Statistical Analysis

Data are presented as mean ± standard deviation. The chi-squared test was employed to evaluate differences in categorical variables, such as clinical characteristics across subgroups. A P-value of < 0.05 was considered statistically significant. The Benjamini-Hochberg method was applied to adjust P-values for multiple comparisons. Data processing, statistical analysis, and visualization were performed using R software (version 4.1.3).

## Results

### Determination of Radiotherapy Resistance Gene Panel

**Figure 1:**
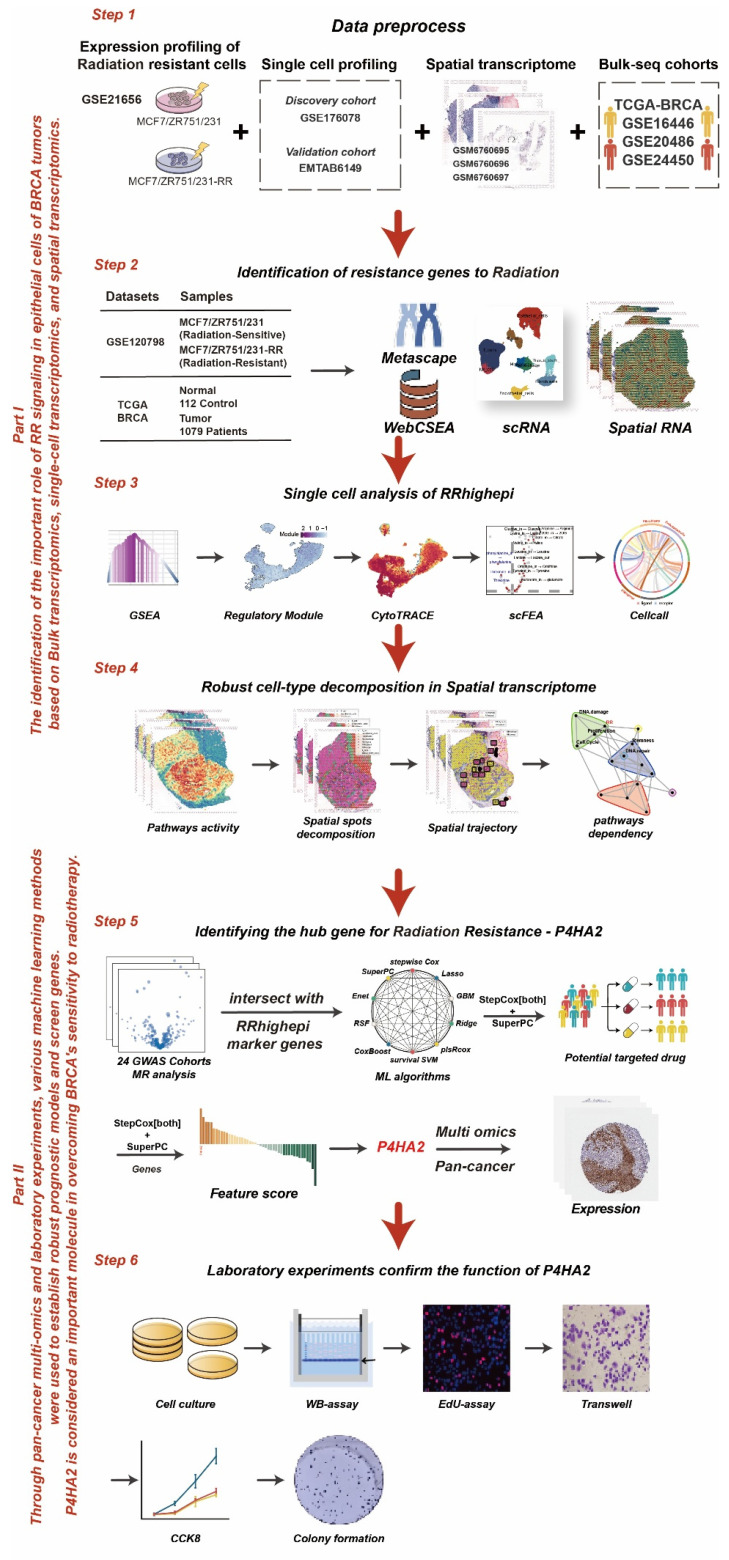
** The workflow of the study**.

**Figure 2:**
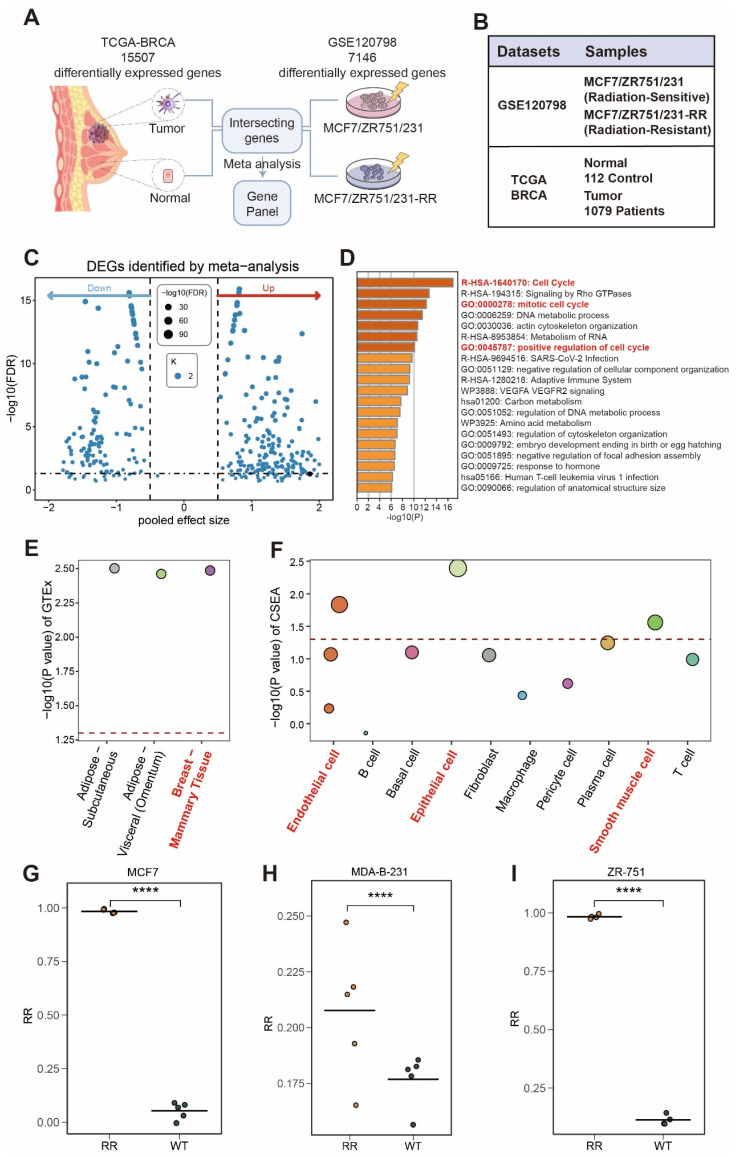
** Identification and function of breast cancer radiotherapy resistance gene panel.** (A) Schematic diagram showing the origin of breast cancer radiotherapy resistance gene panel. (B) Details of the datasets used for gene panel construction. (C) Volcano plot displaying the radiotherapy resistance gene panel. (D) Enrichment analysis of radiotherapy resistance gene panel. (E) Tissue type distribution analysis of radiotherapy resistance gene panel. (F) Cell type distribution analysis of radiotherapy resistance gene panel. (G-I) Comparison of ssGSEA scores of the radiation resistance gene panel between the radiation-resistant group (RR) and the control group (WT) in MCF7, MDAB-231, and ZR751 cell lines.

The study design is depicted in Figure 1. We began by analyzing the TCGA-BRCA and GSE120798 cohorts (Figure 2A), with the corresponding sample details provided in Figure 2B. Differential expression analysis was conducted for both cohorts, and the overlap of upregulated genes in tumor tissues and radiotherapy-resistant groups was identified (Figure 2C, Table S1). Using a threshold of meta_FDR < 0.05 and |meta_Hedges| > 0.7, we obtained a set of significantly differentially expressed genes, which was designated as the "radiotherapy resistance gene panel" (Table S2). Enrichment analysis performed using the Metascape database revealed that this gene panel is predominantly associated with cell cycle-related pathways, including R-HSA-1640170, GO:0000278, and GO:0045787 (Figure 2D). Tissue specificity analysis indicated the highest expression in breast tissue (Figure 2E). Additionally, cell-type specificity analysis using the WebCESA database demonstrated that the gene panel is most prominently expressed in breast epithelial cells, endothelial cells, and smooth muscle cells (Figure 2F). Furthermore, we found that the ssGSEA scores of the radiation resistance gene panel in the radiation-resistant group (RR) were significantly higher than those in the control group (WT) among MCF7, MDAB-231, and ZR751 cell lines (Figure 2G-I).

### Elevated Expression of Radiotherapy Resistance Gene Panel in BRCA Epithelial Tumor Cells

**Figure 3:**
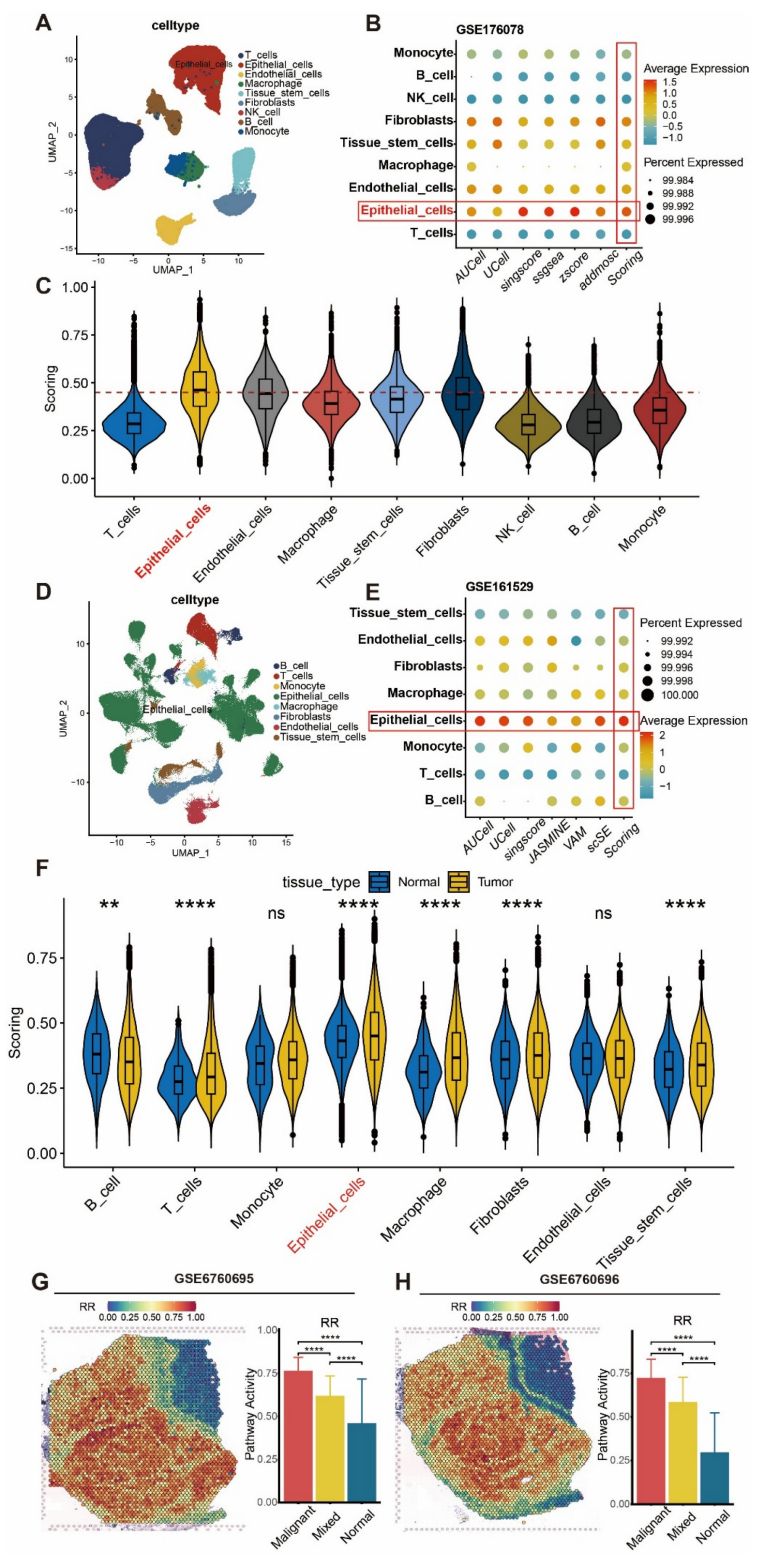
** Elevated expression of radiotherapy resistance gene panel in BRCA tumor epithelial cells.** (A) UMAP visualization of cell types after batch correction, dimensionality reduction, and clustering in GSE176078 dataset. (B) Bubble plot showing radiotherapy resistance gene panel scores across cell types using multiple scoring methods in GSE176078 dataset. (C) Violin plot displaying average enrichment scores across cell types in GSE176078 dataset. (D) UMAP visualization of cell types after batch correction, dimensionality reduction, and clustering in GSE161529 dataset. (E) Bubble plot showing radiotherapy resistance gene panel scores across cell types using multiple scoring methods in GSE161529 dataset. (F) Violin plot displaying average enrichment scores across cell types in GSE161529 dataset. (G-H) Enrichment scores of radiotherapy resistance gene panel in spatial transcriptomics and differential analysis between regions.

To further explore the cell specificity of the radiotherapy resistance gene panel, we analyzed BRCA single-cell cohorts, performing batch correction, dimensionality reduction, clustering, and cell annotation (Figure 3A). Using multiple single-cell scoring methods, we evaluated the radiotherapy resistance gene panel (RR) scores across cell types and compared their average values (Figure 3B-C), finding that BRCA epithelial cells had the highest RR scores. In a validation cohort following batch correction, dimensionality reduction, clustering, and cell annotation (Figure 3D), we confirmed that BRCA epithelial cells exhibited the highest RR scores (Figure 3E), with increased scores in epithelial cells of tumor tissues (Figure 3F). To further investigate RR specificity in tumor tissues, we identified and characterized malignant and benign regions in spatial transcriptomics using the Cottrazm R package (Figure S1A-B), revealing higher RR scores in malignant compared to benign tissues (Figure 3G-H).

**Figure 4:**
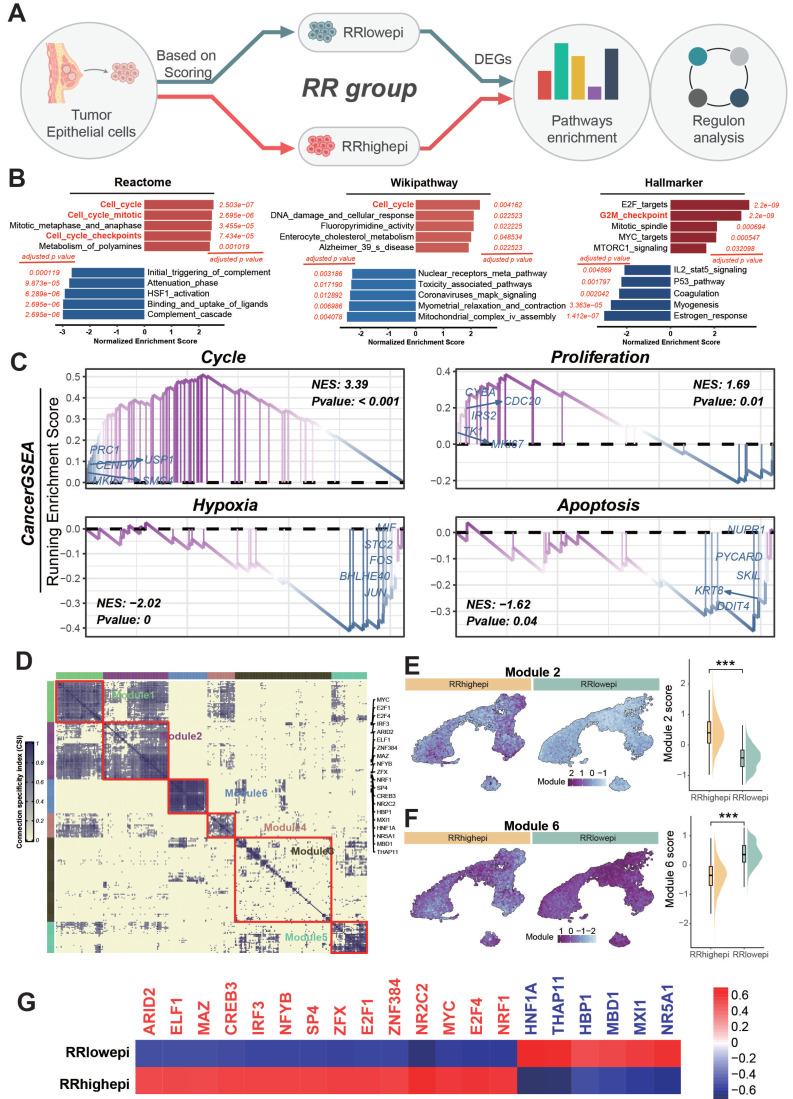
** Identification and functional analysis of RRhighepi cell population.** (A) Schematic diagram of functional identification of RRhighepi cell population. (B) Bar plot showing Reactome, Hallmarker, and KEGG pathway enrichment analysis. (C) Line plot displaying enrichment analysis of tumor-related states/pathways from CancerGSEA. (D) Regulon Module analysis of BRCA tumor epithelial cells. (E-F) Differential comparison of regulon Modules in RR (Radiation resistant) groups. (G) Heatmap showing activities of different transcription factors in RR groups (red font indicates higher activity in RRhighepi, blue font indicates higher activity in RRlowepi).

Based on scoring, we divided epithelial cells into two groups (RR group, median value was 0.450658): high RR score (RRhighepi, RRhighepi group was defined as epithelial cells with a score > 0.450658) and low RR score (RRlowepi, RRlowepi group was defined as epithelial cells with a score ≤ 0.450658) (Figure 4A). Differential enrichment analysis using Reactome, Wikipathway, and Hallmarker pathways revealed upregulation of cell cycle-related pathways in RRhighepi, including cell cycle, cell cycle mitotic, cell cycle checkpoint, and G2M checkpoint (Figure 4B). Additionally, enrichment analysis using 12 tumor states from CancerGSEA showed upregulation of cell cycle and proliferation states in RRhighepi, while hypoxia and apoptosis were downregulated (Figure 4C). To investigate the mechanisms underlying pathway activation, we conducted a transcriptional regulatory analysis on BRCA epithelial cells. We assessed the Connection Specificity Index (CSI) and identified six transcriptional modules (Figure 4D), finding that RRhighepi had higher scores in module 2 (Figure 4E) and lower scores in module 6 (Figure 4F). Transcription factor activity analysis revealed enhanced activity of *ARID2*, *ELF1*, *MAZ*, *CREB3*, *IRF3*, *NFYB*, and *E2F1* in RRhighepi (Figure 4G), which are representative genes of module 2.

### RRhighepi Exhibits Higher Tumor Stemness Characteristics

**Figure 5:**
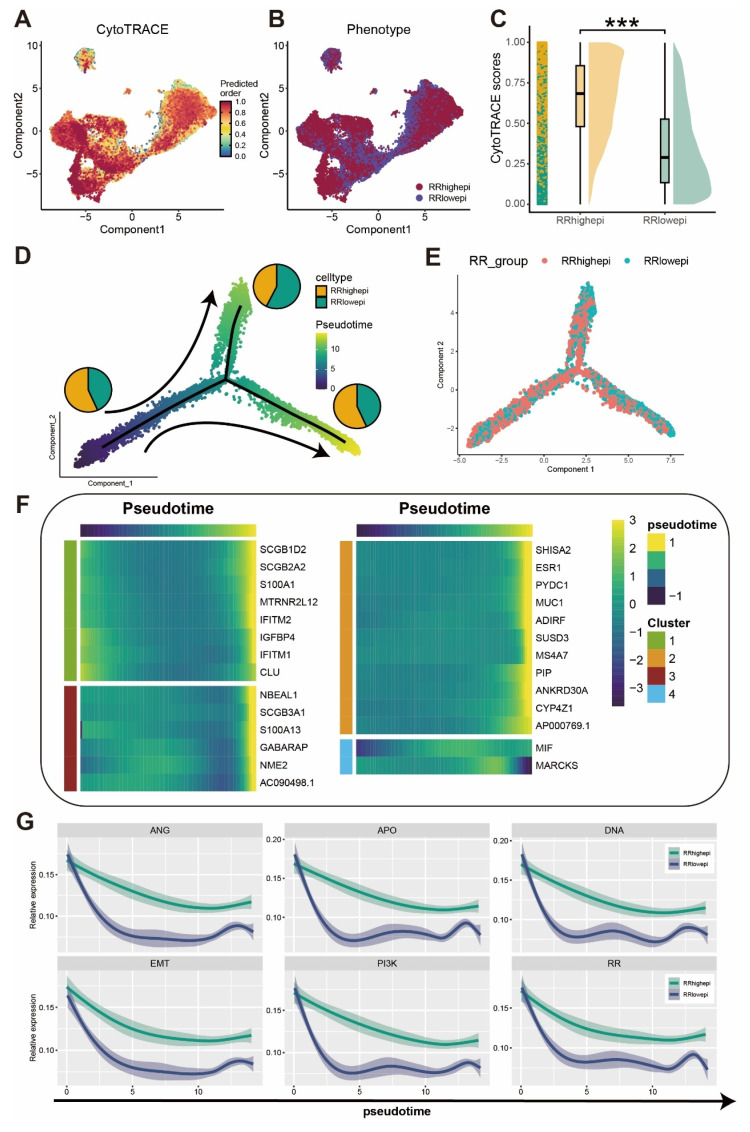
** Single-cell exploration of cellular origin in RR groups.** (A) CytoTRACE analysis of cell differentiation potential in RR groups. (B) UMAP visualization of RR group classification. (C) Raincloud plot showing differential comparison of CytoTRACE scores in RR groups. (D-E) Cell type proportion analysis and grouping display under developmental trajectory. (F) Pseudotemporal analysis of genes related to cell development. (G) Differential comparison of tumor activity-related pathway pseudotemporal analysis in RR groups.

To further investigate the developmental dynamics within RR groups, we conducted pseudotime analysis using the monocle algorithm. Initially, tumor stemness characteristics in the RR groups were inferred using the CytoTRACE algorithm (Figure 5A-B), which revealed higher stemness scores in the RRhighepi group (Figure 5C). Cells with elevated CytoTRACE stemness scores were designated as developmental starting points, and developmental trajectories were inferred using the monocle algorithm. This analysis indicated a decrease in the proportion of RRhighepi cells as development advanced (Figure 5D-E). Cellular development-related genes were grouped into four clusters (Figure 5F), with *ESR1*, *NME2*, and *IGFBP4* showing predominant expression at later developmental stages. A further exploration of tumor-associated pathways, including epithelial-mesenchymal transition (EMT), angiogenesis (ANG), DNA damage repair (DNA), the* PI3K* pathway (PI3K), apoptosis (APO), and radiotherapy resistance gene panels (RR) during the pseudotime analysis of RR groups (Figure 5G), showed a gradual reduction in pathways such as DNA damage repair and the *PI3K* pathway as pseudotime progressed. Notably, RRhighepi consistently demonstrated higher tumor-associated characteristics compared to RRlowepi throughout the pseudotime trajectory.

**Figure 6:**
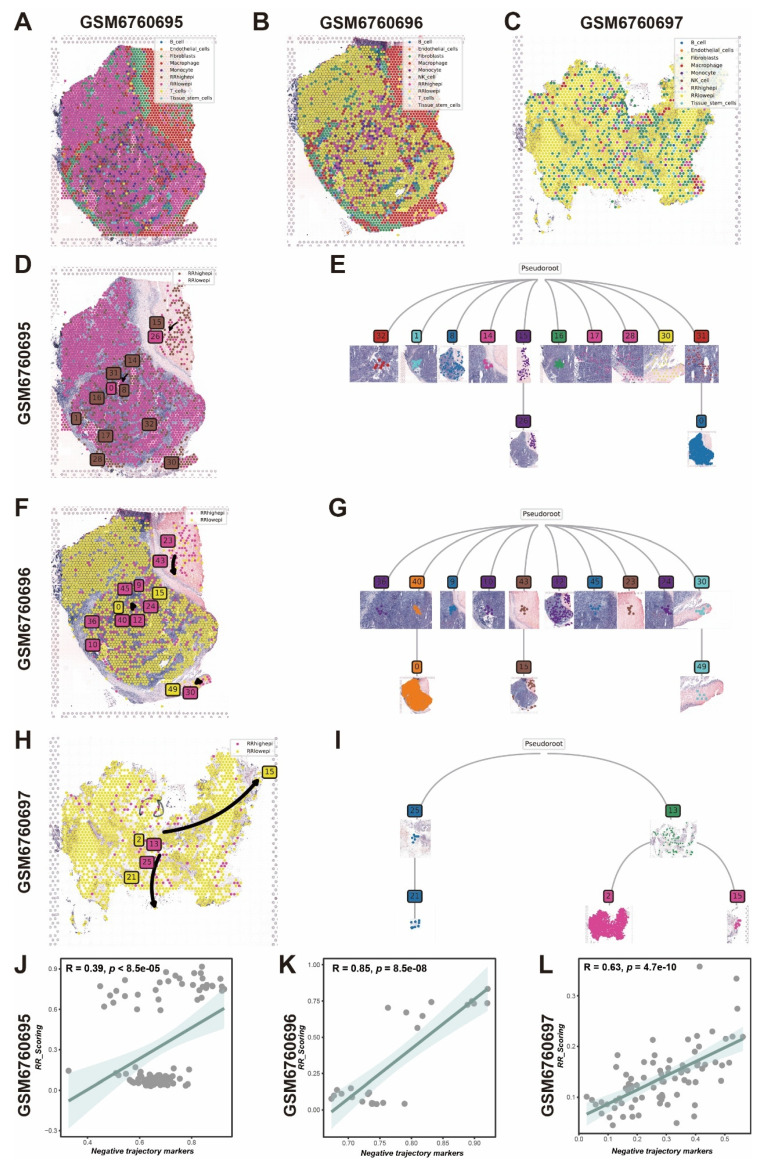
** Spatial transcriptomics exploration of RR group cellular origin.** (A-C) Cell types after spatial transcriptomics deconvolution. (D,E) Cell developmental trajectory and trajectory tree of RR groups in spatial transcriptomics sample GSM6760695. (F,G) Cell developmental trajectory and trajectory tree of RR groups in spatial transcriptomics sample GSM6760696. (H,I) Cell developmental trajectory and trajectory tree of RR groups in spatial transcriptomics sample GSM6760697. (J-L) Correlation scatter plots between radiotherapy resistance gene panel scores and developmental trajectory genes across multiple spatial transcriptomics samples.

Subsequently, we used RCTD deconvolution to map single-cell cohort cell types to spatial transcriptomics (Figure 6A-C) and employed stlearn to infer the developmental trajectory of RR groups. We found that the spatial transcriptomic developmental trajectory proceeds from RRhighepi to RRlowepi, with the developmental trajectory tree illustrating detailed evolutionary relationships between cell clusters (Figure 6D-I). Additionally, we observed that radiotherapy resistance gene panel scores positively correlate with trajectory evolution gene enrichment scores (Figure 6J-L). Based on these findings, we propose that the developmental progression involves a transition from a stem-like, therapy-resistant state (RRhighepi) toward a more differentiated state (RRlowepi), with the latter expanding during this progression.

### Single-Cell Metabolomic Differences in RRgroups

**Figure 7:**
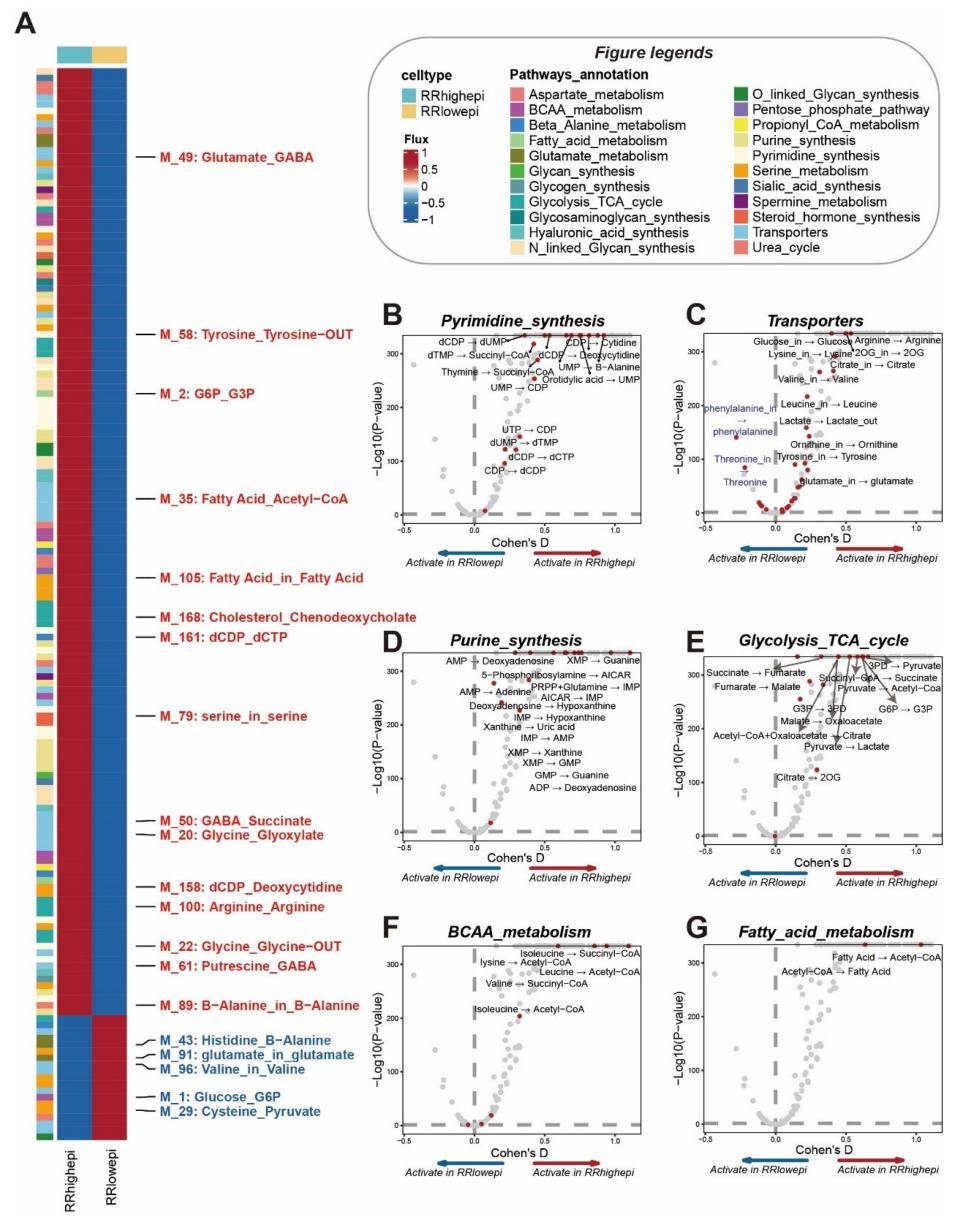
** Single-cell metabolomic differences in RR groups.** (A) Heatmap showing differences in metabolite conversion-related pathways between RR groups (red font indicates higher activity in RRhighepi, blue font indicates higher activity in RRlowepi). (B-G) Cohen's D differential analysis in Pyrimidine synthesis (B), Transporters (C), Purine synthesis (D), Glycolysis TCA cycle (E), BCAA metabolism (F), and Fatty acid metabolism (G).

Our previous findings revealed the upregulation of genes involved in 'Metabolism of polyamines' and 'Enterocyte cholesterol metabolism' pathways in RRhighepi (Figure 3B). Therefore, we used the scFEA algorithm 23 to conduct detailed metabolomic exploration of RR groups. Among 169 metabolic pathways, the majority of upregulated pathways were in RRhighepi, including G6P to G3P conversion, Fatty Acid uptake, serine uptake, and Fatty Acid Acetyl to CoA conversion (Figure 7A). Conversely, upregulated pathways in RRlowepi included Valine uptake and Glucose to G6P conversion (Figure 7A). We further identified multiple activated pathways in RRhighepi related to Pyrimidine synthesis (Figure 7B), Transporters (Figure 7C), Purine synthesis (Figure 7D), Glycolysis TCA cycle (Figure 7E), BCAA metabolism (Figure 7F), and Fatty acid metabolism (Figure 7G), indicating that RRhighepi possesses more robust metabolic characteristics.

### Interaction Between RRhighepi and Endothelial Cells

**Figure 8:**
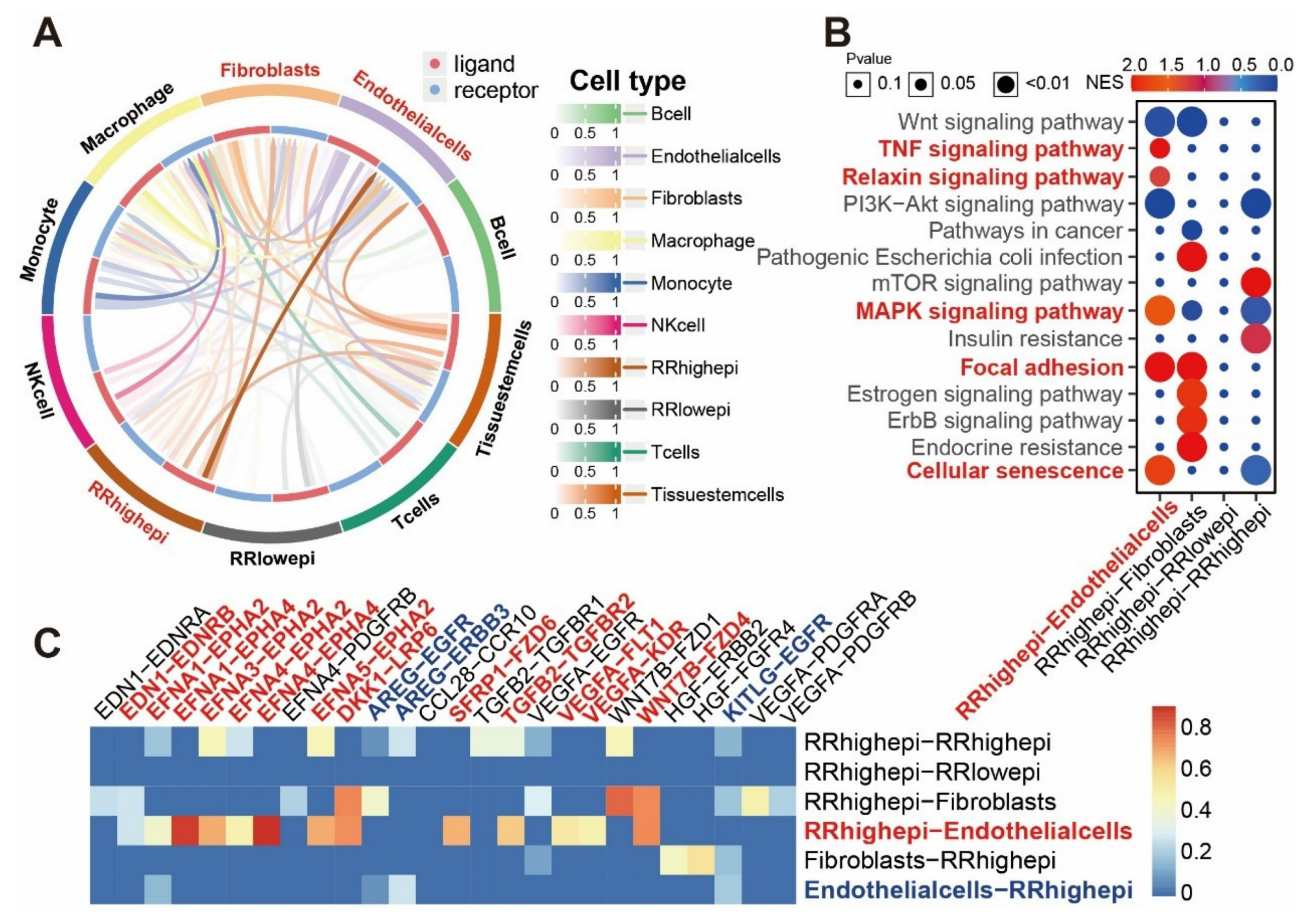
** Interaction between RRhighepi and endothelial cells.** (A) Interaction intensity analysis between RRhighepi and multiple cell types. (B) Analysis of activated pathways in various cell communications. (C) Analysis of activated ligand-receptor pairs in various cell communications.

Given the important role of the tumor microenvironment in radiotherapy, we further explored cell-cell communication between RR groups and other cell types at the single-cell level used the Cellcall algorithm 17. We discovered strong communication intensity between RRhighepi and endothelial cells (Figure 8A), with their interactions primarily activating Cellular senescence, Focal adhesion, MAPK signaling pathway, Relaxin signaling pathway, and TNF signaling pathway (Figure 8B). Analyzing ligand-receptor pairs, we found that the main pairs mediating communication from RRhighepi to endothelial cells included *WNT7B-FZD4, EFNA3-EPHA2, EFNA1-EPHA4*, and *EFNA4-EPHA4* (Figure 8C, Figure S5). From endothelial cells to RRhighepi, key ligand-receptor pairs included *AREG-EGFR, KITLG-EGFR*, and *AREG-ERBB3* (Figure 8C). These findings indicate that RRhighepi communicates with endothelial cells through multiple pathways.

### Dependency of the Radiotherapy Resistance Gene Panel (RR) on Cell Cycle and Tumor States

As identified in our initial analysis (Figure 2D), the radiotherapy resistance gene panel is primarily enriched in cell cycle-related pathways. This finding was further supported by our subsequent spatial transcriptomics analysis. Using data from 12 tumor cell states within the CancerGSEA database, we conducted spatial transcriptomic enrichment analysis to assess the relationship between the radiotherapy resistance gene panel and various tumor states. Dependency analysis of pathways with mistyR 25 revealed that the gene panel is predominantly associated with the cell cycle, DNA damage, DNA repair, and cell proliferation states across regions of spatial colocation, immediate neighborhoods, and extended neighborhoods (15 spots) (Figure S2A-C), with these results confirmed in multiple spatial transcriptomic samples.

By integrating results from spatial transcriptomic deconvolution with tumor state enrichment analysis and applying mistyR for cell-pathway dependency evaluation, we observed that RRhighepi shows a strong reliance on the radiotherapy resistance gene panel (RR), as well as pathways related to the cell cycle, DNA damage, DNA repair, and proliferation in both intra- and para-spatial regions (Figure S2D-F). These findings lead to the conclusion that the radiotherapy resistance gene panel is closely tied to the cell cycle, DNA damage, DNA repair, and other related pathways or states in tumors.

### Mendelian Randomization Analysis of Radiotherapy Resistance Gene Panel and Breast Cancer Causality

**Figure 9:**
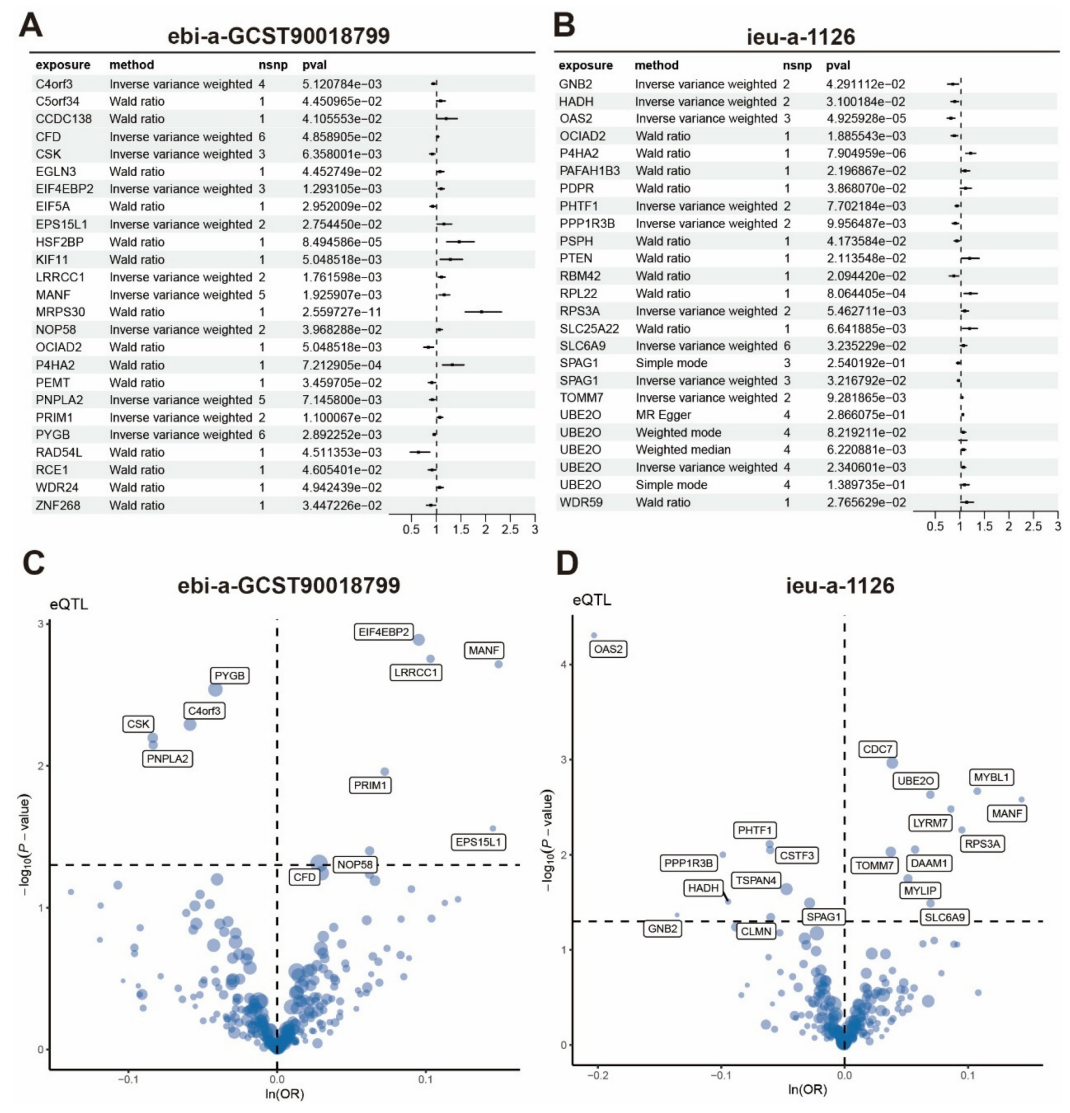
** Mendelian randomization exploration of causal relationships between radiotherapy resistance-related genes and breast cancer.** (A-B) Forest plots showing MR analysis results of radiotherapy resistance-related genes in ebi-a-GCST90018799 cohort and ieu-a-1126 cohort. (C-D) Volcano plots displaying eQTL results of radiotherapy resistance-related genes.

We collected 24 breast cancer-related GWAS cohorts and performed Mendelian randomization to analyze the causal relationship between SNPs in the radiotherapy resistance gene panel and breast cancer GWAS. In the ebi-a-GCST90018799 cohort, genes with causal relationships to breast cancer included *NOP58, OCIAD2, P4HA2, PEMT*, and *PNPLA2* (Figure 9A, C). In the ieu-a-1126 cohort, genes with causal relationships to breast cancer included* GNB2, HADH, OAS2, OCIAD2, P4HA2*, and *PAFAH1B3* (Figure 9B, D). Across 24 GWAS cohorts, we identified 318 genes with causal relationships to breast cancer (Table S3).

### Robust Machine Learning Model for Predicting Survival and Guiding Treatment Decisions

**Figure 10:**
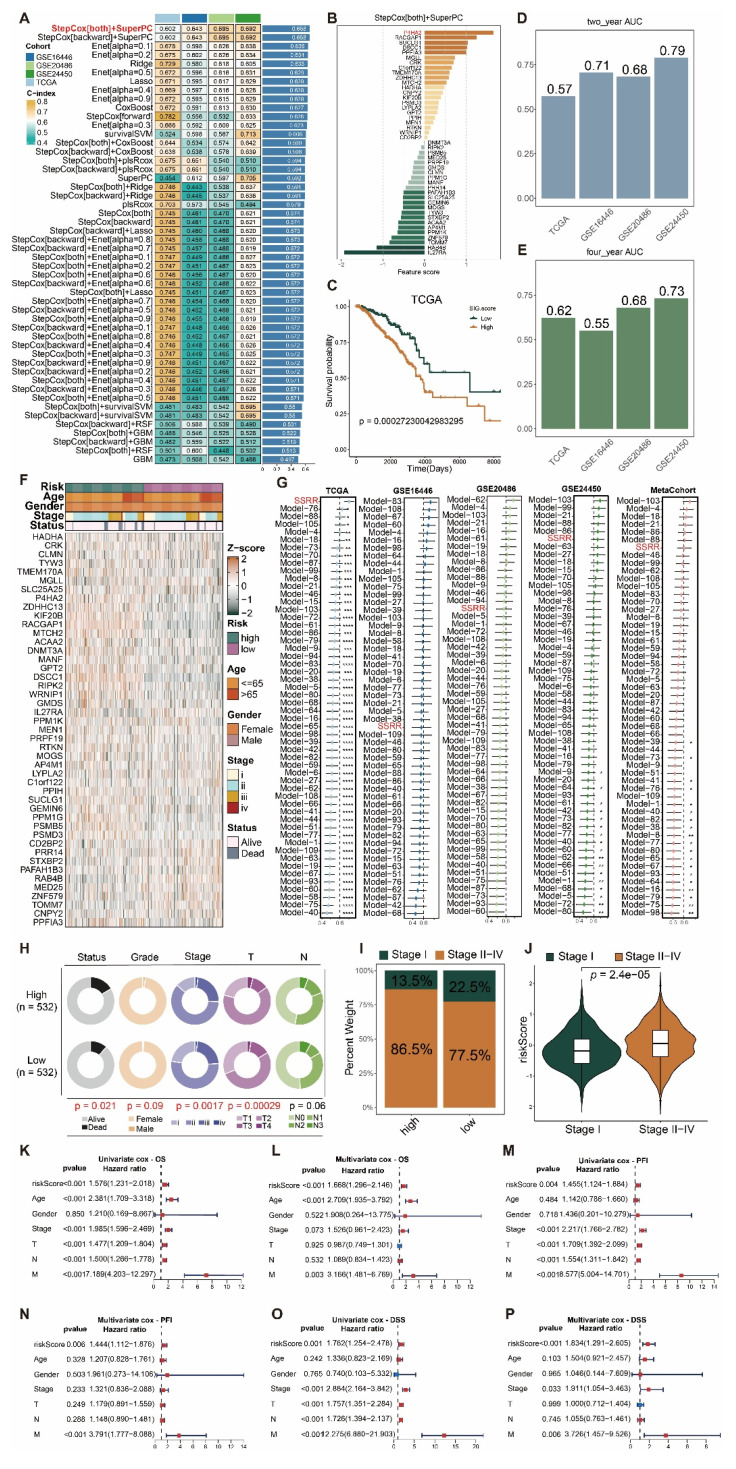
** Establishment of RRhighEpi-related prognostic model and relationship between prognostic score and clinical factors.** (A) Heatmap showing C index of various machine learning approaches for prognostic model construction. (B) Bar plot displaying gene weights under StepCox[both]+SuperPC algorithm. (C) Survival analysis of risk scores under prognostic model assessment in TCGA-BRCA cohort. (D-E) AUC values for 2-year and 4-year survival across cohorts using StepCox[both]+SuperPC method for prognostic model construction. (F) Heatmap showing expression of RRhighepi signature genes in risk score groups in TCGA-BRCA cohort. (G) Horizontal comparison of prognostic models constructed using StepCox[both]+SuperPC method. (H) Impact of risk score on patient survival, tumor grade, tumor stage, and TNM classification. (I-J) Relationship between risk score and tumor grade. (K-P) Univariate and multivariate analysis of risk score and clinical indicators' impact on OS, PFI, and DSS.

To identify signature genes for RRhighepi (|logFC|>0.25) (Table S4), we used the Findmarker function and cross-referenced these genes with those from Mendelian randomization analysis (Figure S3) to construct a prognostic model (Table S5). Among the various machine learning methods tested for predicting patient survival, StepCox[both]+SuperPC consistently ranked in the top three based on average c-index (Figure 10A), maintaining stable gene weights (Figure 10B). As a result, we adopted this method to develop our prognostic model, named SSRR (SuperPC and StepCox-based Radiotherapy Resistance model). By using the median SSRR risk score as a threshold, patients were stratified into high-risk and low-risk groups. In the TCGA cohort, the high-risk group demonstrated significantly worse survival outcomes compared to the low-risk group (Figure 10C). The area under the curve (AUC) values for two-year survival in the TCGA, GSE16446, GSE20486, and GSE24450 cohorts were 0.57, 0.71, 0.68, and 0.79, respectively, while for four-year survival, the AUC values were 0.62, 0.55, 0.68, and 0.73, respectively. Expression patterns of the SSRR weight genes are illustrated in Figure 10F, with *P4HA2*, *KIF20B*, and *DSCC1* showing higher expression in the high-risk group.

When comparing SSRR to other prognostic models, SSRR consistently ranked among the top models in c-index across independent cohorts (Figure 10G). Stratifying patients into high-risk and low-risk groups based on the median SSRR value and analyzing the relationship with clinical indicators revealed significant differences in survival status, tumor grade, tumor stage, and T stage between the groups (Figure 10H). The high-risk group had a higher proportion of stage II-IV tumors than the low-risk group (Figure 10I), and higher SSRR risk scores were observed in stage II-IV tumors compared to stage I tumors (Figure 10J). To determine whether SSRR is an independent prognostic factor for BRCA, univariate and multivariate Cox regression analyses were performed on OS, PFI, and DSS in the TCGA-BRCA dataset. The results indicated that SSRR was a significant risk factor for OS, PFI, and DSS in univariate analysis (HR>1, p<0.001). Additionally, in multivariate analysis, SSRR remained an independent prognostic factor for OS (HR:1.476, p<0.001), PFS (HR:1.471, p < 0.001), and DSS (HR:1.780, p < 0.001) (Figure 10K-P), demonstrating its strong prognostic capability in BRCA patients.

**Figure 11:**
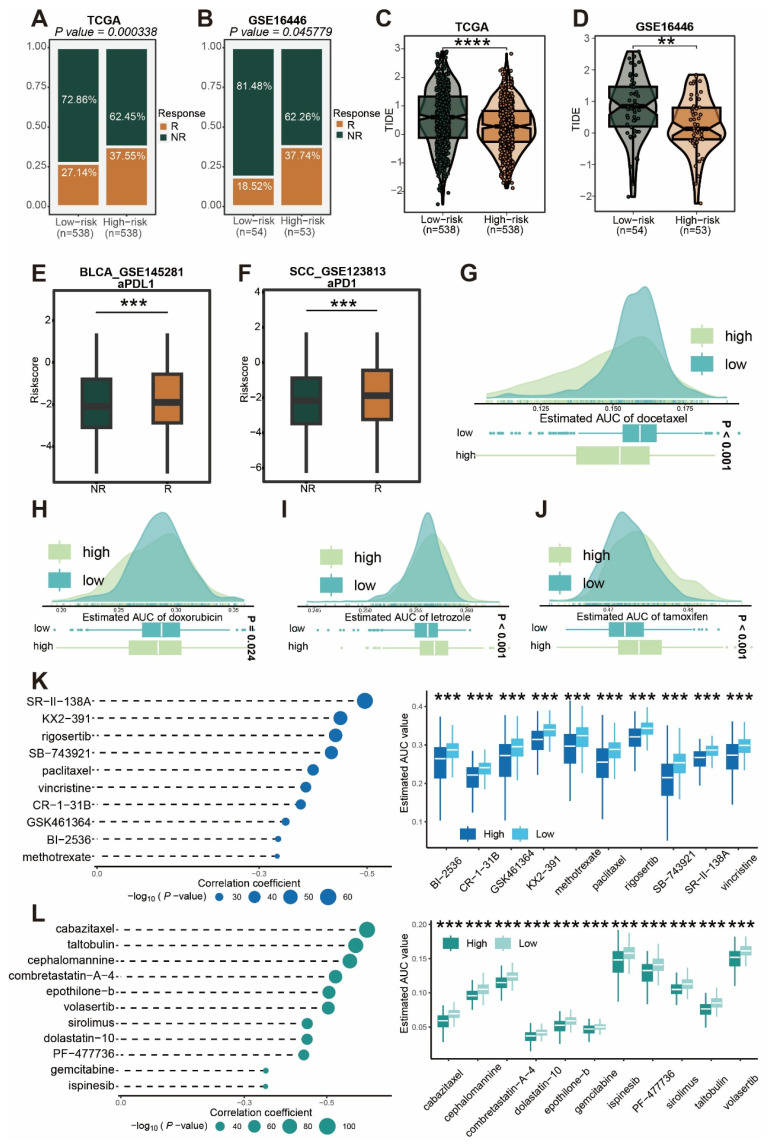
** Relationship between risk score and clinical treatment.** (A-B) Prediction of immunotherapy response in high and low risk score groups in TCGA and GSE16446 cohorts. (C-D) Comparison of TIDE scores in high and low risk score groups in TCGA and GSE16446 cohorts. (E-F) Relationship between risk score and treatment response in immunotherapy single-cell cohorts BLCA_GSE145281 (E) and SCC_GSE123813 (F). (G-J) Drug sensitivity analysis for docetaxel (G), doxorubicin (H), letrozole (I), and tamoxifen (J) in high and low risk score groups. (K-L) Analysis of potential small molecule drugs for high and low risk score groups.

To further examine differences in immunotherapy response between high-risk and low-risk groups, we applied the Submap algorithm to predict immunotherapy outcomes in the TCGA and GSE16446 cohorts. The high-risk group showed a better response to immunotherapy (Figure 11A-B), while the low-risk group exhibited higher TIDE scores (Figure 11C-D). Additionally, analysis of public single-cell immunotherapy datasets revealed that cells responsive to immunotherapy had higher risk scores (Figure 11E-F). Given the poor prognosis of patients in the high-risk group, we conducted an analysis of the CTRP and PRISM databases and found that this group was more sensitive to drugs such as docetaxel and doxorubicin (Figure 11G-H), while the low-risk group showed greater sensitivity to drugs like letrozole and tamoxifen (Figure 11I-J). Furthermore, we identified potential drugs for the high-risk group, including KX2-391, rigosertib, cabazitaxel, and taltobulin (Figure 11K-L).

### *P4HA2* as a Potential Therapeutic Target for Breast Cancer

**Figure 12:**
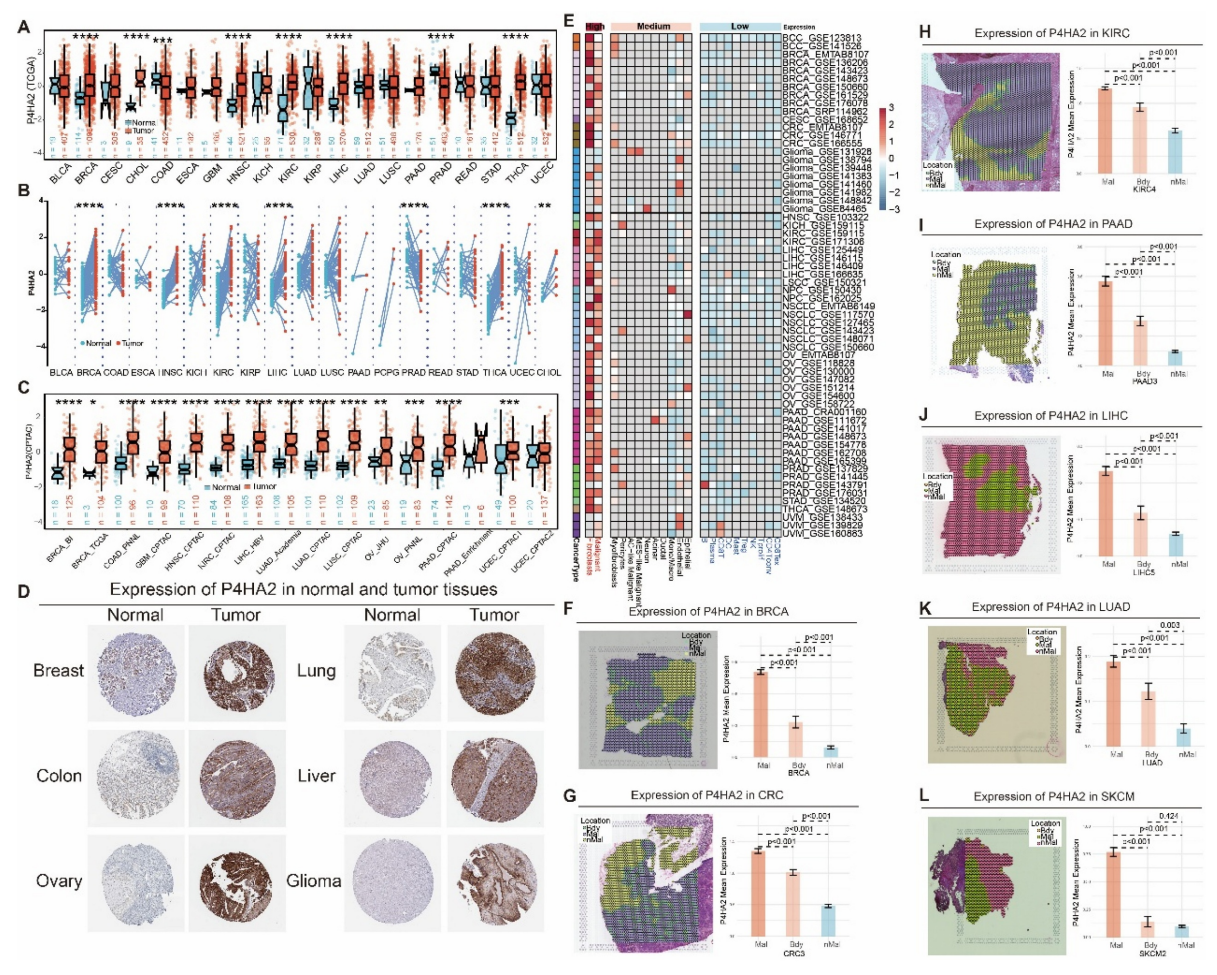
** Expression differences of *P4HA2* in normal tissues and various tumor tissues.** (A) mRNA expression levels of *P4HA2* in normal and tumor samples across TCGA pan-cancer cohorts (including paired and unpaired samples). (B) mRNA expression levels of *P4HA2* in normal and tumor samples in paired samples from TCGA pan-cancer cohorts. (C) Protein expression levels of *P4HA2* in normal and tumor samples in CPTAC pan-cancer proteotranscriptomic cohorts. (D) Immunohistochemical staining levels of *P4HA2* in various normal and tumor tissues. (E) Heatmap showing mRNA expression levels of *P4HA2* across different cell types in pan-cancer single-cell cohorts. (F-L) Expression levels of *P4HA2* in spatial transcriptomics samples of BRCA (F), CRC (G), KIRC (H), PAAD (I), LIHC (J), LUAD (K), and SKCM (L).

Analysis of SSRR model genes revealed that *P4HA2* had the highest weight (Figure 10B). We conducted in-depth exploration of *P4HA2* at the transcriptomic, proteomic, and genomic levels across pan-cancer datasets. In TCGA transcriptomics, *P4HA2* expression was elevated in multiple cancers (BRCA, CHOL, HNSC, KIRC, LIHC, THCA) and decreased in PRAD (Figure 12A). Similar findings were observed in paired samples: *P4HA2* expression was increased in BRCA, HNSC, KIRC, LIHC, and THCA, while decreased in PRAD (Figure 12B). In CPTAC proteomics data, *P4HA2* protein expression was elevated in BRCA, HNSC, KIRC, LIHC, and unlike mRNA expression, also in COAD, GBM, LUAD, LUSC, OV, PAAD, and UCEC, suggesting mechanisms involved in transcriptional and translational regulation (Figure 12C). Additionally, immunohistochemical staining showed higher *P4HA2* levels in Breast, Lung, Colon, Liver, Ovary, and Glioma tissue sections compared to normal tissues (Figure 12D). In pan-cancer single-cell transcriptomics, *P4HA2* showed higher expression in malignant tumor cells and fibroblasts, with lower expression in B cells and T cells (Figure 12E). Furthermore, in spatial transcriptomics of multiple cancers (BRCA, CRC, KIRC, PAAD, LIHC, LUAD, SKCM), *P4HA2* expression was consistently higher in malignant regions compared to non-malignant regions (Figure 12F-L).

**Figure 13:**
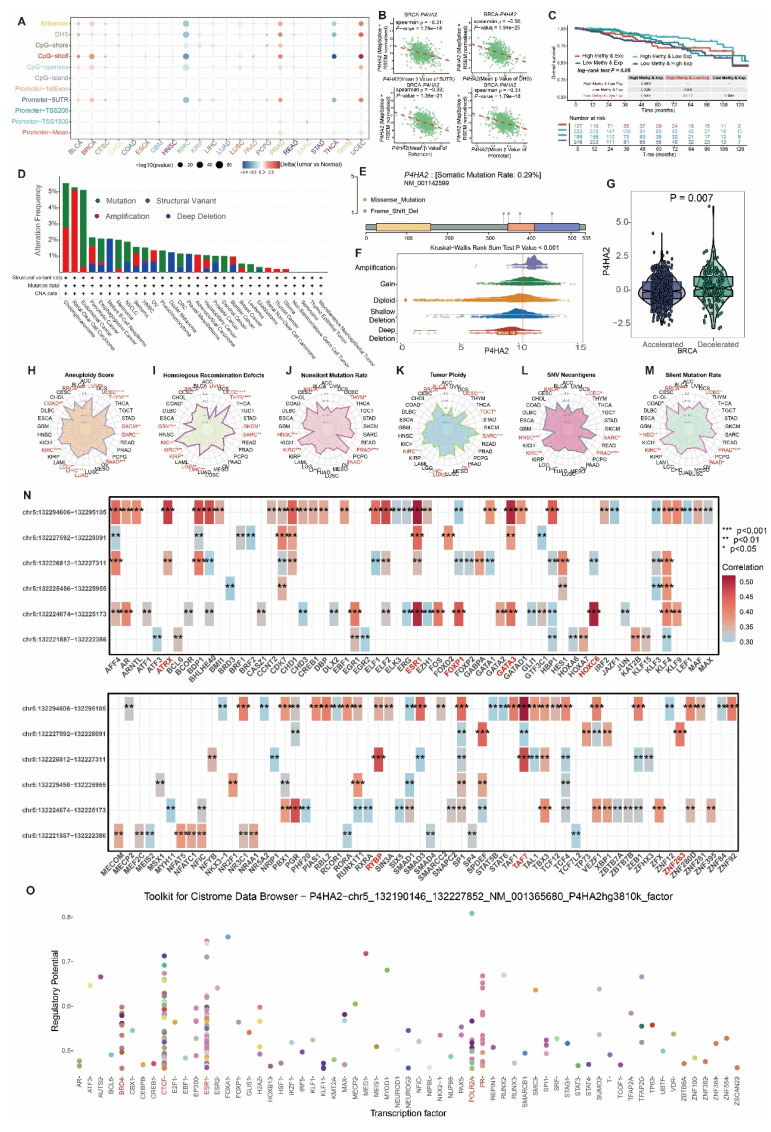
** Integrated analysis of *P4HA2*-related multi-omics molecular characteristics and clinical phenotypes, and prognostic evaluation.** (A) Differences in *P4HA2* methylation levels across pan-cancer. (B) Spearman correlation between average *P4HA2* gene expression and methylation at different sites in BRCA. (C) Disease-free survival curves for four different subgroups based on DNA methylation (Methy) and gene expression (Exp) levels. (D) Differences in mutation frequency of *P4HA2* across different mutation types in pan-cancer. (E) Distribution of mutation sites of *P4HA2* across different samples. (F) Differences in *P4HA2* expression across different CNV types in pan-cancer. (G) Differences in *P4HA2* expression between biological age acceleration/deceleration groups in breast cancer (BRCA). (H-M) Spearman correlation between different gene set enrichment scores (aneuploidy, homologous recombination deficiency, tumor ploidy, single nucleotide variant neoantigens, non-silent mutation rate, silent mutation rate) and *P4HA2* expression across pan-cancer. (N) Spearman correlation analysis of *P4HA2* gene ATAC-Peak and transcription factors. (O) Prediction of potential transcriptional regulatory factors for *P4HA2*.

Further exploration of *P4HA2* genomics revealed positive correlation between methylation levels and expression in BLCA, BRCA, PRAD, and UCEC, but negative correlation in KIRC and THCA (Figure 13A). Focusing on BRCA methylation, we found negative correlation between expression and methylation across different regions (5UTR, DHS, Enhancer, Promoter) (Figure 13B). Regarding survival, BRCA patients with high methylation levels showed significantly better survival compared to those with low methylation levels (Figure 13C). In terms of mutation frequency, *P4HA2* showed mutation rates above 5% in Cholangiocarcinoma, Renal Clear Cell Carcinoma, and Endometrial Cancer, with Renal Clear Cell Carcinoma predominantly showing Amplification, Endometrial Cancer showing Mutation, while Breast cancer had mutation frequency below 1% (Figure 13D). Across multiple samples, the NM_001142599 transcript of *P4HA2* had a mutation frequency of 0.29%, primarily Missense Mutations and Frame Shift Deletions (Figure 13E). *P4HA2* expression varied significantly across mutation types, with highest expression in Amplification and Gain groups, and lowest in the DeepDeletion group (Figure 13F). Additionally, *P4HA2* showed higher expression in the biological aging group (Figure 13G). Analysis of genomic instability-related gene sets in pan-cancer transcriptomics revealed positive correlation between *P4HA2* and aneuploidy, homologous recombination deficiency, tumor ploidy, single nucleotide variant neoantigens, non-silent mutation rate, and silent mutation rate in BRCA (Figure 13H-L). In ATAC-Peak and transcription factor correlation analysis, *P4HA2* showed strong correlation with transcription factors *ESR1, ATRX, FOXP1, GATA3, HOXC6, RYBP, TAF7,* and *ZNF263* (Figure 13N), while transcription factor prediction based on the Cistrome Data Browser database identified *BRD4, CTCF, ESR1, POLR2A,* and *PR* as potential transcriptional regulators of *P4HA2* (Figure 13O).

**Figure 14:**
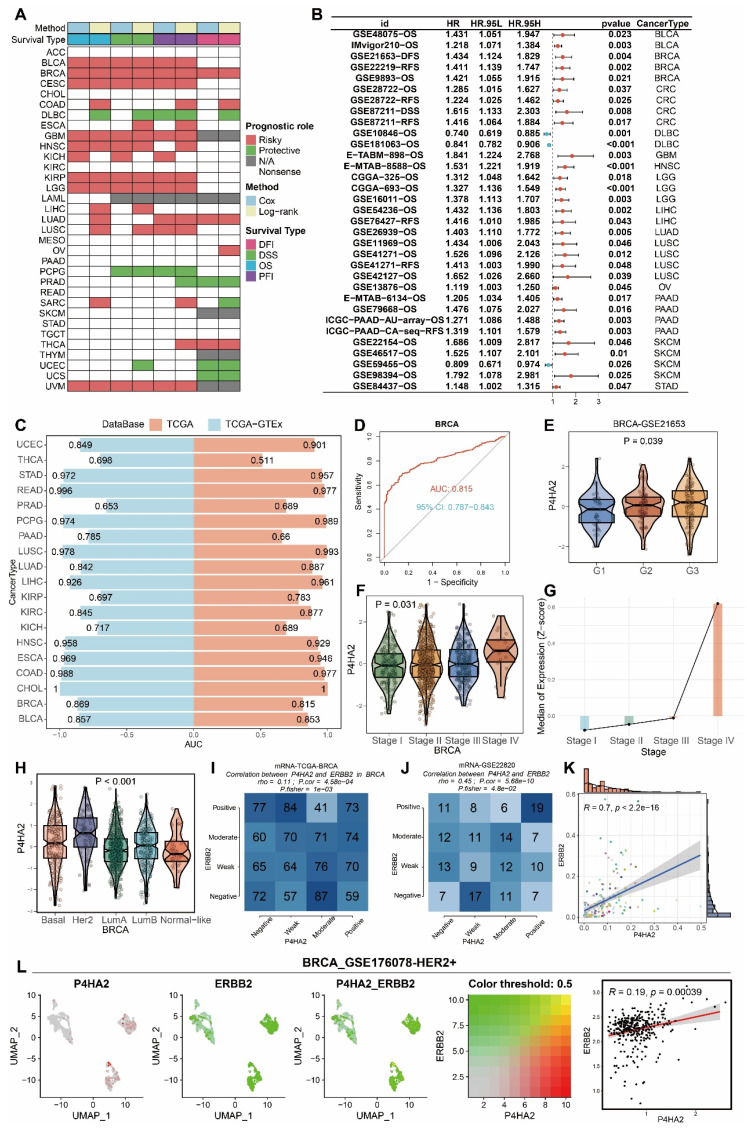
** Prognostic value of *P4HA2* and its correlation with ERBB2.** (A) Relationship of *P4HA2* with different survival periods (disease-free interval DFI, disease-specific survival DSS, overall survival OS, progression-free interval PFI) across pan-cancer. (B) Relationship between *P4HA2* gene expression level and overall survival (OS), disease-free survival (DFS), progression-free survival (PFS), and recurrence-free survival (RFS) assessed by univariate Cox survival analysis across multiple external datasets. (C) Diagnostic performance of *P4HA2* expression in distinguishing tumor from normal groups across pan-cancer. (D) ROC curve showing diagnostic performance of *P4HA2* gene in breast tumors versus normal tissues. (E) Expression differences of *P4HA2* under tumor grading in GSE21653. (F) Expression differences of *P4HA2* gene across different tumor stages (Stage I-IV) in TCGA-BRCA cohort. (G) Median expression (Z-score) across stages. (H) Expression differences of *P4HA2* gene across different molecular subtypes in TCGA-BRCA cohort. (I) Correlation analysis and Fisher's exact test of *P4HA2* and *ERBB2* in mRNA-TCGA-BRCA dataset. (J) Correlation analysis and Fisher's exact test of *P4HA2* and *ERBB2* in mRNA-GSE22820 dataset. (K) Correlation scatter plot analysis of *P4HA2* and *ERBB2*. (L) Correlation of *P4HA2* and *ERBB2* in single-cell cohort.

Next, we analyzed the prognostic value of *P4HA2*. *P4HA2* was found to be a risk factor for OS, DSS, DFI, and PFI in BRCA, a risk factor for OS, DSS, and PFI in BLCA, CESC, GBM, HNSC, KICH, KIRP, LGG, and UVM, but a protective factor for OS, DSS, DFI, and PFI in DLBC (Figure 14A). In univariate COX analysis across multiple cohorts, *P4HA2* was a risk factor for most cancers but potentially a protective factor in DLBC (Figure 14B). Regarding diagnostic performance, *P4HA2* demonstrated AUC values above 0.9 in distinguishing tumor tissues from normal tissues in COAD, HNSC, ESCA, LIHC, LUSC, PCPG, READ, and STAD (Figure 14C), with an AUC of 0.815 in BRCA (Figure 14D). In the GSE21653 breast cancer cohort, *P4HA2* expression showed significant differences across tumor grades, with highest expression in G3 (Figure 14E), while in the TCGA-BRCA cohort, *P4HA2* expression differed significantly across tumor stages, with highest expression in stage 4 (Figure 14F-G). Across breast cancer subtypes, *P4HA2* expression varied significantly, with highest expression in HER2 subtype and lowest in Normal-like subtype (Figure 14H). In TCGA-BRCA and GSE22820 breast cancer cohorts, *P4HA2* expression positively correlated with *ERBB2* (Figure 14I-K). Additionally, in the GSE176078-HER2 positive single-cell cohort, *P4HA2* positively correlated with *ERBB2* (Figure 14L).

**Figure 15:**
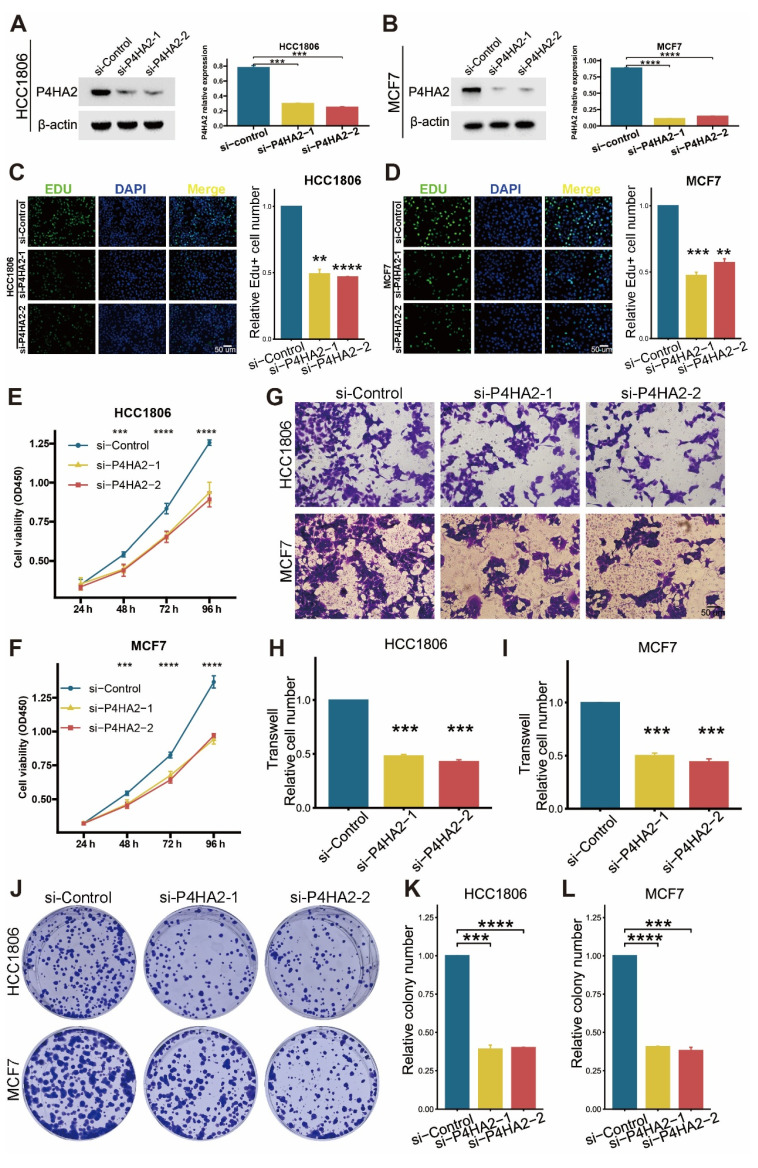
***P4HA2* knockdown significantly inhibits proliferation, invasion, and clone formation ability of HCC1806 and MCF7 cells.** (A-B) Western blot detection of *P4HA2* knockdown efficiency in HCC1806 and MCF7 cells. (C-D) EdU experiment and bar graph showing that *P4HA2* knockdown significantly inhibits DNA replication in HCC1806 and MCF7 cells. (E-F) CCK8 experiment confirming that *P4HA2* knockdown significantly reduces growth ability of HCC1806 and MCF7 cells. (G-I) Transwell invasion experiment and bar graph showing that *P4HA2* knockdown significantly reduces migration ability of HCC1806 and MCF7 cells. (J-L) Clone formation experiment and bar graph showing that *P4HA2* knockdown significantly reduces the number of clones formed by HCC1806 and MCF7 cells. All the above biological experiments were repeated at least three times.

Finally, we conducted laboratory experiments to explore the biological function of *P4HA2*. We used a pair of small interfering RNAs (siRNAs) to downregulate *P4HA2* expression in HCC1806 and MCF7 cell lines. Western blot analysis 48 hours post-transfection confirmed successful knockout of *P4HA2* (Figure 15A-B). After intervention, EdU assays showed reduced cell proliferation in both HCC1806 and MCF7 cell lines (Figure 15C-D). We also observed decreased cell viability (Figure 15E-F). Furthermore, invasion assays demonstrated significantly reduced migration ability in HCC1806 and MCF7 cells after *P4HA2* knockdown (Figure 15G-I). Colony formation assays showed that *P4HA2* downregulation inhibited cell clone formation ability (Figure 15J-L). These results collectively indicate that *P4HA2* promotes proliferation and migration in breast cancer cells.

**Figure 16:**
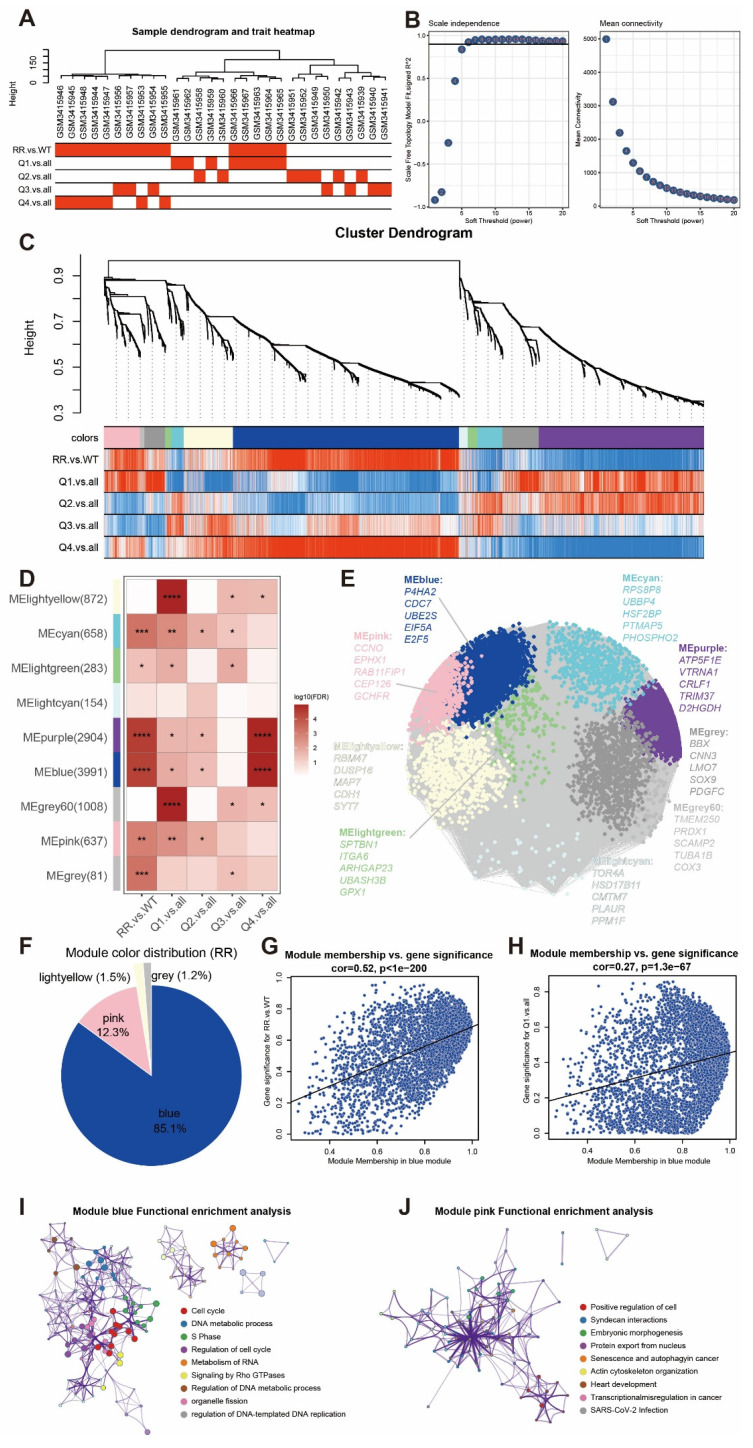
** WGCNA analysis of radioresistance gene panel.** (A) Sample dendrogram and trait heatmap of GSE120798. (B) Scale independence test and mean connectivity analysis of soft threshold. (C) Gene cluster dendrogram. (D) Module-trait correlation heatmap. (E). Module eigengene network diagram. (F) Pie chart of module distribution related to radioresistance gene panel traits. (G) Scatter plot of "module membership" and "gene significance (for radioresistance trait)" of genes in the blue module. (H) Scatter plot of "module membership" and "gene significance (for Q1 vs. all trait)" of genes in the blue module. (I) Functional enrichment analysis network of the blue module. (J) Functional enrichment analysis network of the pink module.

To further elucidate the relationship between *P4HA2* and the radioresistance gene panel, we performed WGCNA analysis on GSE120798. We first conducted sample clustering and soft-threshold analysis (Figure 16A-B), followed by modular clustering of genes (Figure 16C). We found that modules such as MEblue, MEpurple, MEpink, and MEgrey showed strong correlations with radioresistance (Figure 16D), and genes among different modules exhibited complex interaction relationships (Figure 16E). Subsequently, module composition analysis of the radioresistance gene panel revealed that the blue module accounted for 85.1% of the total genes in the radioresistance gene panel, followed by pink (12.3%), light yellow (1.5%), and grey (1.2%) (Figure 16F). Within the blue module, scatter plots showed positive correlations between "module membership" and both "gene significance (for radioresistance trait)" (Figure 16G, cor = 0.52, p < 1e-200) and "gene significance (for *P4HA2* high expression trait)" (Figure 16H, cor = 0.27, p = 1.3e-67), suggesting that genes with high module membership also possess high trait significance. STRING network protein-protein interaction analysis demonstrated strong interactions between *P4HA2* and the radioresistance gene panel (Figure S4). Functional enrichment analysis indicated that the blue module (Figure 16I) was enriched in biological processes including cell cycle, DNA metabolic processes, and regulation of DNA-templated DNA replication; the pink module (Figure 16J) was enriched in pathways including positive regulation of cells, syndecan interactions, and protein export from the nucleus. These results suggest regulatory relationships exist between the radioresistance gene panel and *P4HA2* in relation to radioresistance characteristics.

**Figure 17:**
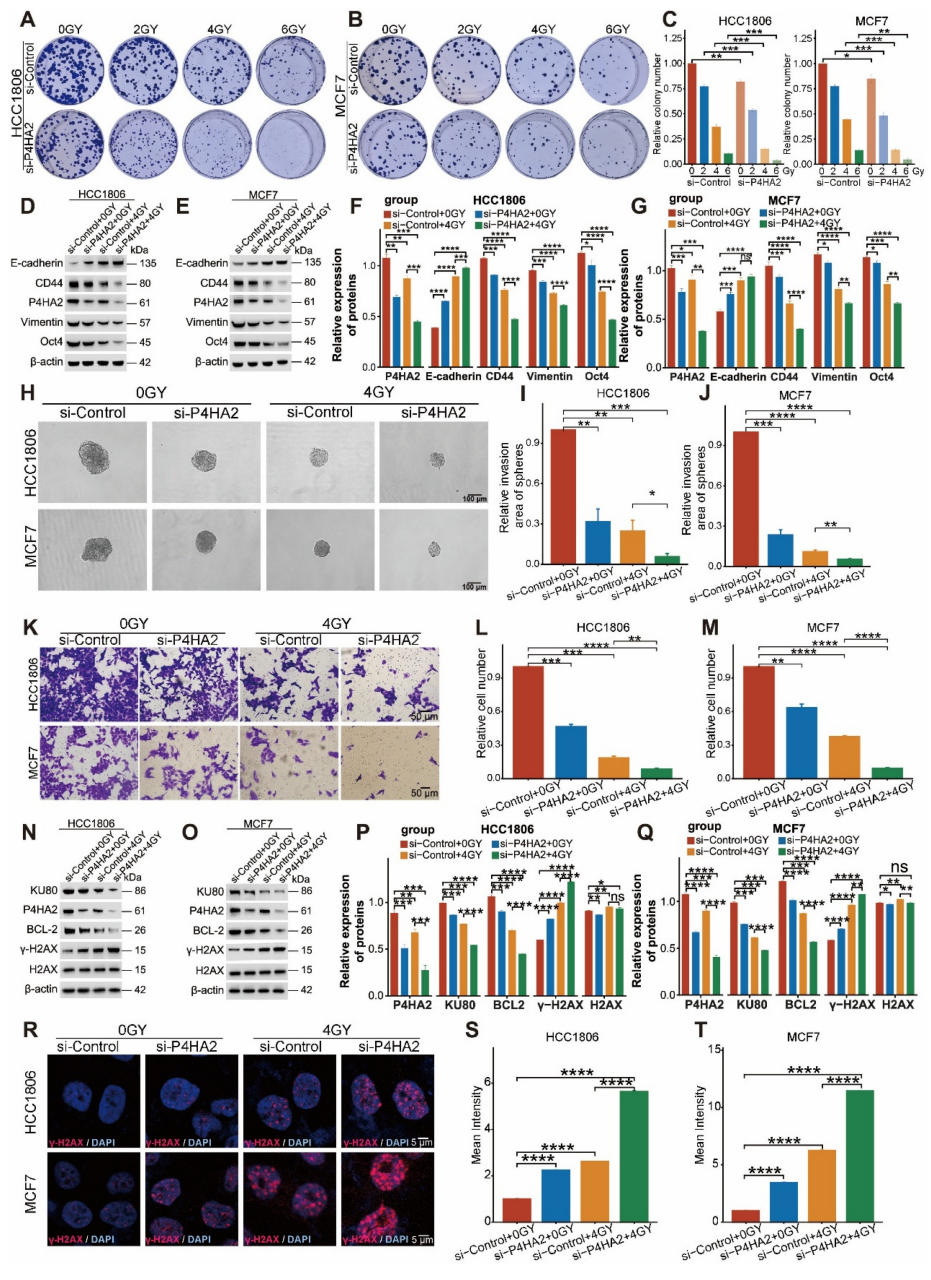
** Effects of *P4HA2* knockdown combined with radiotherapy on breast cancer cells.** (A-C) Clone formation assays show the effects of the combination of multiple radiation doses and *P4HA2* knockdown on breast cancer cells. (D-G) Western blotting (WB) shows the effects of *P4HA2* knockdown combined with radiotherapy on the cancer stemness and EMT characteristics of breast cancer cells. (H-J) Sphere formation assay in suspension shows the effects of *P4HA2* knockdown combined with radiotherapy on the cancer stemness characteristics of breast cancer cells. (K-M) Transwell assay shows the effects of *P4HA2* knockdown combined with radiotherapy on the migration ability of breast cancer cells. (N-Q) Western blotting (WB) shows the effects of *P4HA2* knockdown combined with radiotherapy on the DNA damage characteristics of breast cancer cells. (R-T) Immunofluorescence assay shows the effects of *P4HA2* knockdown combined with radiotherapy on *γ-H2AX* in breast cancer cells. All the above biological experiments were repeated at least three times.

Subsequently, we further explored the effects of radiotherapy and *P4HA2* on breast cancer cells through experimental validation. First, using multiple radiation doses combined with *P4HA2* knockdown, we found that breast cancer cells were significantly reduced under 4 Gy irradiation combined with *P4HA2* knockdown (Figure 17A-C), while cells nearly disappeared under 6 Gy irradiation combined with *P4HA2* knockdown. Using 4 Gy irradiation combined with *P4HA2* knockdown, we found that protein expression levels of *CD44*, *Vimentin*, and *Oct4* in the 4 Gy irradiation plus *P4HA2* knockdown group were significantly lower than in the irradiation-only or *P4HA2* knockdown-only groups, while *E-cadherin* protein expression was significantly increased (Figure 17D-G). Sphere formation assays showed that sphere volume was significantly reduced in the 4 Gy irradiation plus *P4HA2* knockdown group (Figure 17H-J), and Transwell assays demonstrated that cell migration capacity was significantly decreased in the 4 Gy irradiation plus *P4HA2* knockdown group (Figure 17K-M). Furthermore, we found that protein expression levels of *KU80* and* BCL-2* in the 4 Gy irradiation plus *P4HA2* knockdown group were significantly lower than in the irradiation-only or *P4HA2* knockdown-only groups, while *γ-H2AX* protein expression was significantly elevated (Figure 17N-Q). Immunofluorescence showed that the mean fluorescence intensity of *γ-H2AX* was significantly enhanced in the 4 Gy irradiation plus *P4HA2* knockdown group (Figure 17R-T). Taken together, these results suggest that knockdown of *P4HA2* may be a potential target for enhancing radiosensitivity.

## Discussion

Radioresistance represents a major challenge in the treatment of breast cancer (BRCA) 31, 32, particularly under the complex influences of tumor heterogeneity and the tumor microenvironment 33-39. In this study, we identified a gene panel associated with radiotherapy resistance and elucidated its mechanistic role in breast cancer cells through multi-level analyses. Systematic analyses of two independent cohorts, TCGA-BRCA and GSE120798, revealed core features of the resistance gene panel and further explored its potential mechanisms concerning cell specificity, metabolic characteristics, tumor microenvironment, and immune response.

Initially, differential expression analysis was performed on both bulk datasets, TCGA-BRCA and GSE120798, successfully identifying genes closely associated with radiotherapy resistance. Enrichment analyses indicated that these genes predominantly participated in cell cycle-related pathways, including cell cycle regulation, G1/S checkpoint, and G2/M checkpoint pathways. Research indicates that tumor cells can enter a radiation-tolerant persistent state through processes like cell cycle progression and division, temporarily resisting radiotherapy and ultimately promoting tumor regrowth 40. G1/S and G2/M checkpoints are crucial for cell cycle control and can be targeted to counteract radiation-induced damage 41. Further analysis showed that the expression of the radiotherapy resistance gene panel was significantly elevated in breast epithelial cells, suggesting an important role of tumor epithelial cells in radioresistance. Normal cells, upon DNA damage, arrest at the G1 phase via *p53* regulators to initiate DNA repair. Conversely, tumor cells, due to dysfunctional G1/S checkpoints, tend to proceed into S phase and evade repair. Post-radiation, cancer cells activate CAD (Caspase-Activated DNAse), leading to DNA fragmentation and G2 arrest, providing additional time for repair. Inhibiting CAD has been shown to increase radiosensitivity, and targeting G2 checkpoint pathways might enhance radiotherapy efficacy 42-44.

To further explore cell-type specificity, we analyzed single-cell BRCA datasets, revealing that the resistance gene panel was markedly overexpressed in epithelial cells. Within tumor tissues, the resistance scores derived from this panel were significantly higher in epithelial cells, highlighting their potential role in mediating radioresistance. This suggests that radioresistance may be intricately linked to epithelial cell characteristics, especially proliferative states. The concept of tumor cells adopting an adaptive, persistent state to survive and proliferate post-radiation 40, resembling "radiation-tolerant persister cells" (RTP cells), was further examined via viral budding simulation methods, which facilitated tumor re-proliferation.

Subsequently, spatial transcriptomics analyses validated these findings by revealing that the resistance gene panel was more highly expressed in malignant regions of breast cancer tissues compared to benign regions. This supports the notion that resistance-related genes are involved in the formation of malignant tumor areas, with resistant cancer cells likely persisting within these regions and contributing to tumor recurrence.

Enrichment analyses indicated that cell cycle pathways were significantly upregulated in the radioresistant epithelial cell subgroup (RRhighepi), including pathways related to the cell cycle and G2/M checkpoint. This aligns with earlier observations that cell cycle control is a key mechanism of radiotherapy resistance in breast cancer, notably through the activation of the G2/M checkpoint, which functions as a negative regulator of cell cycle progression 41. The activation of cell cycle arrest in radioresistant epithelial cells effectively induces a state of proliferation blockade. Upon radiation-induced DNA damage, tumor cells engage cell cycle arrest, further activating self-repair mechanisms and evading radiation injury, thereby increasing resistance 42. However, residual tumor cells often re-enter a proliferative state during tumor repopulation 45. Studies suggest that cell death induced by radiochemotherapy can generate potent growth-stimulatory signals, promoting tumor regeneration 45, 46. Moreover, transcription factor activity analysis revealed elevated activity of several transcription factors, such as *ARID2, E2F1, E2F4, NFYB*, and *MYC*, in the RRhighepi group, all of which are closely associated with cell proliferation and tumor progression. E2F1, for instance, can induce transcription of genes involved in cell cycle entry and apoptosis regulation 47. *NFYB*-mediated upregulation of *E2F1* enhances *CHK1* signaling, contributing to chemotherapy resistance in colorectal cancer 48. Therefore, the proliferation and radioresistance of epithelial cells in the context of radiotherapy may be mediated by overactivation of cell cycle-related genes.

Metabolic pathway analysis via single-cell metabolomics further revealed that activation of multiple metabolic routes-including fatty acid metabolism, amino acid metabolism, glycolysis, pyrimidine synthesis, and purine synthesis-is prominent in the RRhighepi group. These metabolic adaptations provide essential energy support and likely sustain proliferation and survival of resistant cells 49-52. It should be noted that the analysis of pathways such as polyamine and cholesterol metabolism in this study is based on computational inference from transcriptomic data. While this provides valuable hypotheses, it does not directly measure metabolite levels or flux. Future studies employing targeted or untargeted metabolomics techniques on sorted cell populations will be essential to experimentally confirm these metabolic alterations. Furthermore, the observed metabolic differences between RR groups may reflect not only intrinsic programming disparities but also potential influences of feedback mechanisms, such as the inhibition of biosynthetic pathways due to sufficient end-product levels. Studies demonstrate that radiation enhances glycolysis in pancreatic cancer cells, raising lactate levels and promoting MDSC activity via the *GPR81/mTOR/HIF-1A/STAT3* pathways, thus contributing to an immunosuppressive microenvironment and tumor progression, recurrence, and radioresistance 53. Tumor cells undergo metabolic reprogramming, including increased mitochondrial fatty acid oxidation (FAO), which supplies ATP through mitochondrial breakdown to aid escape from radiation-induced cell death. FAO also upregulates *CD47* expression via the citrate-Acetyl-CoA-RelA pathway, contributing to immune suppression and protecting radioresistant GBM cells from macrophage-mediated phagocytosis 54-56. Additionally, increased purine levels in glioblastoma enhance DNA repair, further promoting resistance 57. These findings underscore the significance of metabolic reprogramming in tumor cell survival under radiotherapy and highlight the importance of understanding these mechanisms to develop combination therapies that can improve treatment outcomes.

Further investigations into the interaction between the resistance gene panel and tumor microenvironment, especially with endothelial cells, revealed that RRhighepi influences intercellular communication pathways. Radiotherapy can modulate endothelial functions to promote tumor progression through mechanisms such as EndMT (Endothelial-to-Mesenchymal Transition), which increases the expression of stem cell markers like *CD44v6*, associated with increased osteopontin secretion in lung cancer 58. Additionally, radiation enhances tumor-associated macrophage (TAM) M2 polarization 58. In hepatocellular carcinoma, irradiated endothelial cells facilitate tumor invasion through cytokine secretion changes and extracellular matrix (ECM) remodeling 59, 60. Notably, doses of 2 Gy rather than higher doses like 6 Gy elicited these pro-invasive responses, indicating that radiation dosage and scheduling may modulate tumor-stroma interactions and influence therapy efficacy 59, 60. Radiation also augments endothelial drug efflux capacity, contributing to blood-brain barrier-mediated drug resistance 61. Based on single-cell communication analyses, we observed significant signaling interactions between RRhighepi and endothelial cells primarily via ligand-receptor pairs such as *WNT7B-FZD4* and *EFNA3-EPHA2*. *WNT7B* activates β-catenin signaling pathways involved in HPV E6-induced tumor angiogenesis in cervical cancer 62 and promotes gastric cancer progression via *WNT7B-m6A-TCF7L2* feedback loops 63. The *EFNA3/EPHA2* axis modulates cellular metabolic plasticity, promoting stemness features in hypoxic hepatocellular carcinoma 64. These pathways play critical roles in cell migration, proliferation, and tumor microenvironment remodeling, suggesting that RRhighepi may foster tumor angiogenesis and invasion through these signaling networks, thereby enhancing tumor growth and metastatic potential.

Through various analytical approaches, the expression of *P4HA2* was found to be significantly elevated in breast cancer, correlating closely with tumor stage, grade, and patient prognosis. *P4HA2* is markedly overexpressed across multiple cancer types, especially in BRCA. Laboratory experiments confirmed that silencing *P4HA2* suppressed proliferation and migration abilities of breast cancer cells. We further found that *P4HA2* knockdown can also exert a synergistic effect with radiotherapy - the combination of 4 Gy radiotherapy and *P4HA2* knockdown significantly reduced the number of breast cancer cells, while almost eliminating these cells when combined with 6 Gy irradiation, which fully demonstrates knockdown of P4HA2 in enhancing radiosensitivity. Mechanistically, WGCNA analysis revealed that *P4HA2* is closely associated with the core modules of the radiation resistance gene panel (e.g., the MEblue module, which accounts for 85.1% of the panel genes). Moreover, module membership showed positive correlations with gene significance for both radiation resistance and *P4HA2* overexpression, suggesting that *P4HA2* may function by regulating radiation resistance-related pathways such as cell cycle and DNA metabolism. String interaction analysis further validated the functional crosstalk between *P4HA2* and the radiation resistance gene panel. In functional experiments, *P4HA2* knockdown combined with radiotherapy not only decreased the expression of cancer stemness markers (e.g., *CD44*, *Oct4*) and inhibited sphereforming and migration capacities, but also impaired DNA damage repair and anti-apoptotic abilities by downregulating *KU80* and *BCL-2* while upregulating *γ-H2AX*. Although our functional studies did not involve direct isolation of the RRhighepi subpopulation, the concurrent suppression of stemness markers (e.g., *Oct4*) and tumorsphere formation, coupled with enhanced radiosensitivity following *P4HA2* knockdown, provides strong correlative evidence that the stem-like state contributes to the resistant phenotype. Future studies utilizing specific cell surface markers for RRhighepi cell sorting would be valuable to directly test their radiotherapeutic response. Our experimental results demonstrating that *P4HA2* knockdown suppresses proliferation, and migration are consistent with previously reported findings that *P4HA2*-mediated collagen prolyl hydroxylation regulates cancer cell plasticity and migration 65. Our study extends this knowledge by establishing a direct link between *P4HA2* and radiotherapy resistance, demonstrating its role in enhancing DNA damage repair and maintaining a stem cell-like state upon radiation exposure. Interventions targeting *P4HA2* are expected to provide a novel strategy for breast cancer treatment, particularly holding significant translational value for cases with radiotherapy resistance.

In conclusion, this comprehensive multi-layered bioinformatics analysis reveals the biological significance of the radiotherapy resistance gene panel in breast cancer and its potential clinical applications. The panel exerts its effects through mechanisms such as cell cycle regulation, metabolic reprogramming, and tumor microenvironment interactions, thereby augmenting tumor cell proliferation and survival. Importantly, *P4HA2* emerges as a potential therapeutic target with significant prognostic value in the context of radiotherapy resistance. Future therapeutic strategies focusing on the inhibition of this gene panel and its associated pathways may pave the way for personalized treatment approaches in breast cancer. Nevertheless, further experimental validation and clinical trials are essential to evaluate these findings' translational potential and their relevance in overcoming radiotherapy tolerance and resistance in breast cancer.

## Supplementary Material

Supplementary figures and table legends.

Supplementary tables.

## Abbreviations

RR: Radiotherapy resistance
RRhighepi: RRhigh epithelial cells
TCGA: The Cancer Genome Atlas
BRCA: Breast invasive carcinoma
P4HA2: Prolyl 4-Hydroxylase Subunit Alpha 2
HER2: Human epidermal growth factor receptor 2
ER: Estrogen receptor
TNBC: Triple-negative breast cancer
TME: The tumor microenvironment
EMT: Epithelial-Mesenchymal Transition
