# Supplementary material for: Single-cell and spatial transcriptomics reveal P4HA2-mediated radiotherapy resistance mechanisms in breast cancer
Source: Theranostics. 2026 Jan 1;16(5):2192–220. doi: 10.7150/thno.121257 (PMC12712805; doi:10.7150/thno.121257)
Supplement: Supplementary file 1 — Supplementary figures and table legends. [file thnov16p2192s1.pdf]

Single-cell and spatial transcriptomics reveal P4HA2-mediated radiotherapy resistance mechanisms in breast cancer

Huimin Li[1,2†], Junzhi Liu[2†], Yuheng Jiao[3†], Fengyu Xu[1], Shurui Wang[1,4]*, Qiang Tang[1]*

[1] The Second Affiliated Hospital of Zhejiang University School Medicine, Hang Zhou, 310000, China.

[2] Department of Oncology, Shanghai Pulmonary Hospital, School of Medicine, Tongji University, Shanghai, 200433, China.

[3] Department of Heart failure, Shanghai East Hospital, School of Medicine, Tongji University, Shanghai, 200120, China.

[4] Chinese Academy of Medical Sciences & Peking Union Medical College, Beijing 100730, China.

†These authors contributed equally to this work.

*** Correspondence:**
tangqiang678@zju.edu.cn (Qiang Tang);

Supplementary Materials Legends

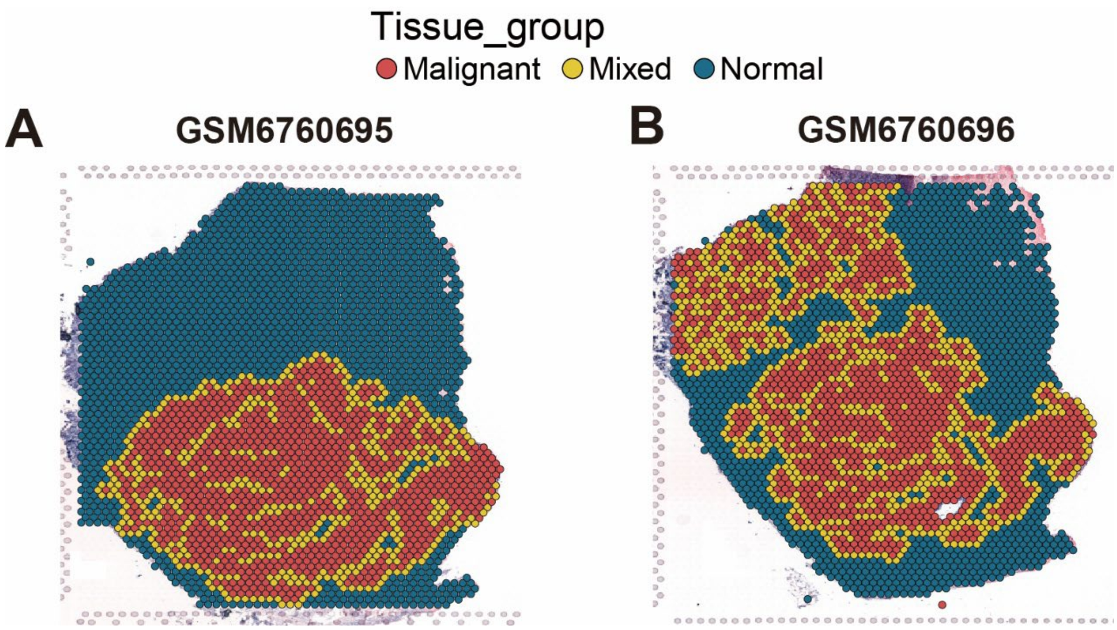

Figure S1. Identification and characterization of benign and malignant regions in spatial transcriptomics based on R package Cottrazm. (A-B) Delineation of benign and malignant regions in spatial transcriptomics.

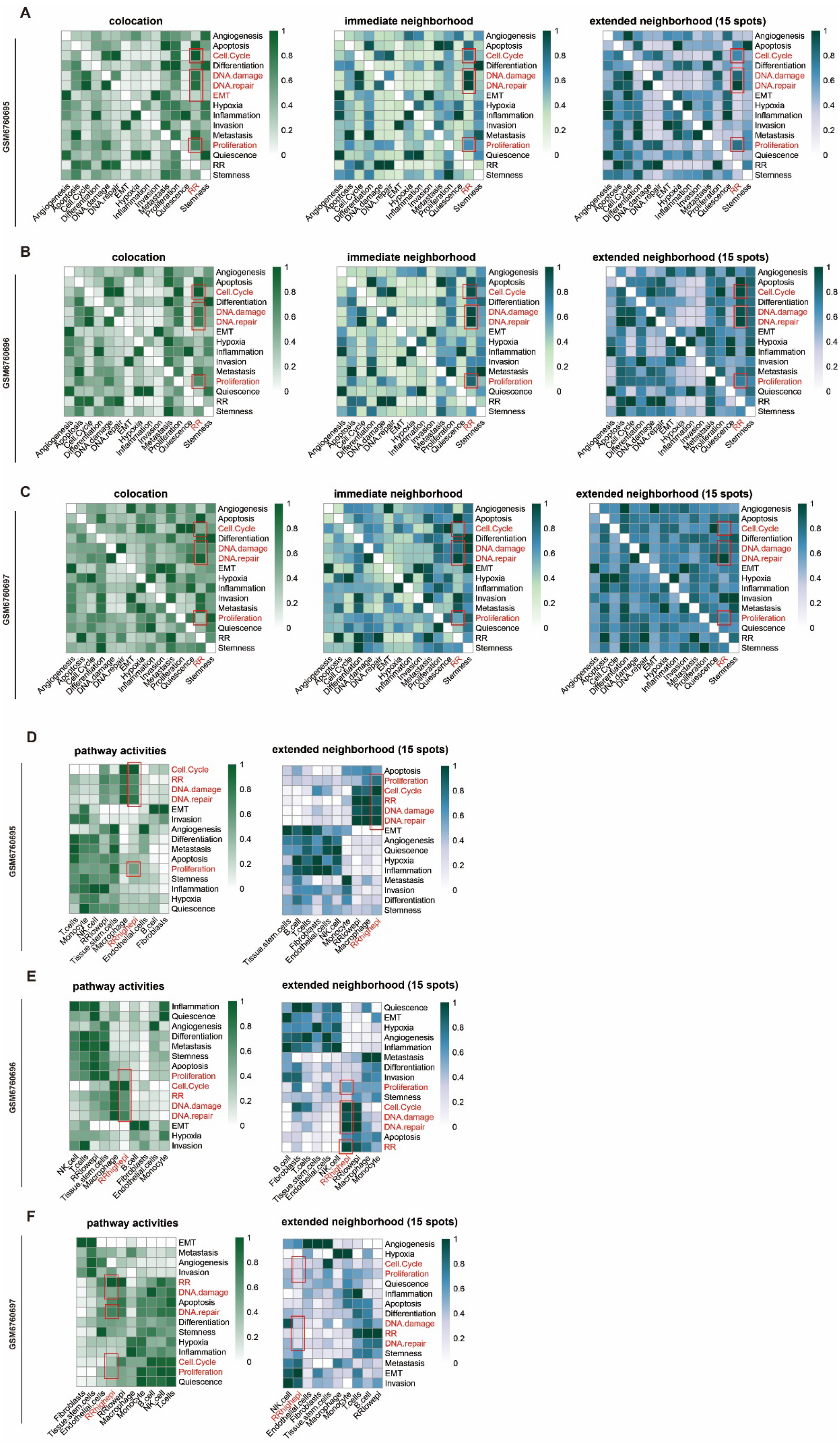
A
colocation
immediate neighborhood
extended neighborhood (15 spots)
GSM6760695
Angiogenesis
Apoptosis
Cell.Cycle
Differentiation
DNA.damage
DNA.repair
EMT
Hypoxia
Inflammation
Invasion
Metastasis
Proliferation
Quiescence
RR
Stemness
B
colocation
immediate neighborhood
extended neighborhood (15 spots)
GSM6760696
C
colocation
immediate neighborhood
extended neighborhood (15 spots)
GSM6760697
D
pathway activities
extended neighborhood (15 spots)
GSM6760695
T.cells
Monocyte
NK.cell
RRlowepi
Tissue.stem.cells
Macrophage
RRhighepi
Endothelial.cells
B.cell
Fibroblasts
E
pathway activities
extended neighborhood (15 spots)
GSM6760696
F
pathway activities
extended neighborhood (15 spots)
GSM6760697

Figure S2. Cell and pathway dependency analysis in spatial transcriptomics. (A-C) Heatmap showing pathway-pathway dependency analysis in spatial transcriptomics colocation, immediate neighborhood, and extended neighborhood (15 spots) regions. (D-F) Heatmap showing cell-pathway dependency analysis in spatial transcriptomics intra and para regions.

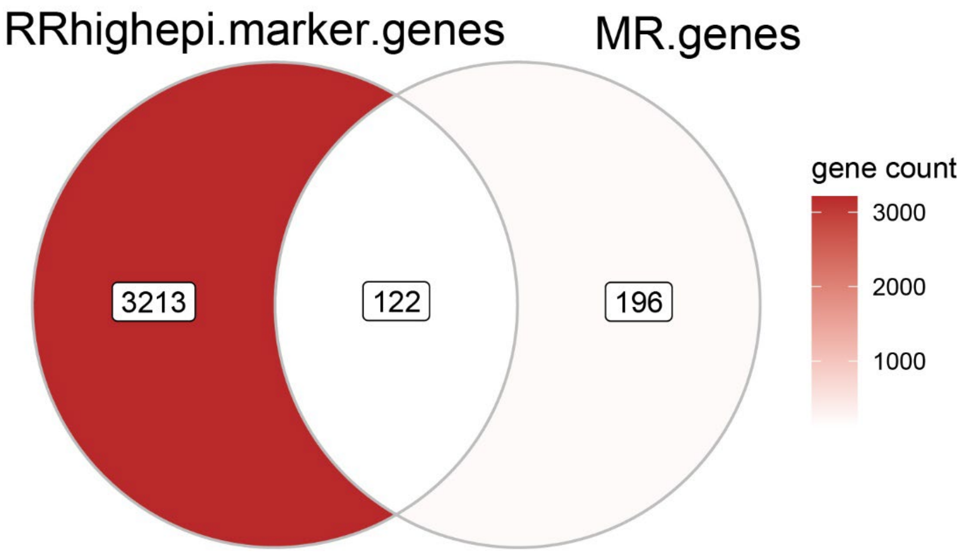

Figure S3. Intersection of RRhighepi genes and genes filtered by Mendelian randomization to obtain genes for establishing prognostic model (ML.genes).

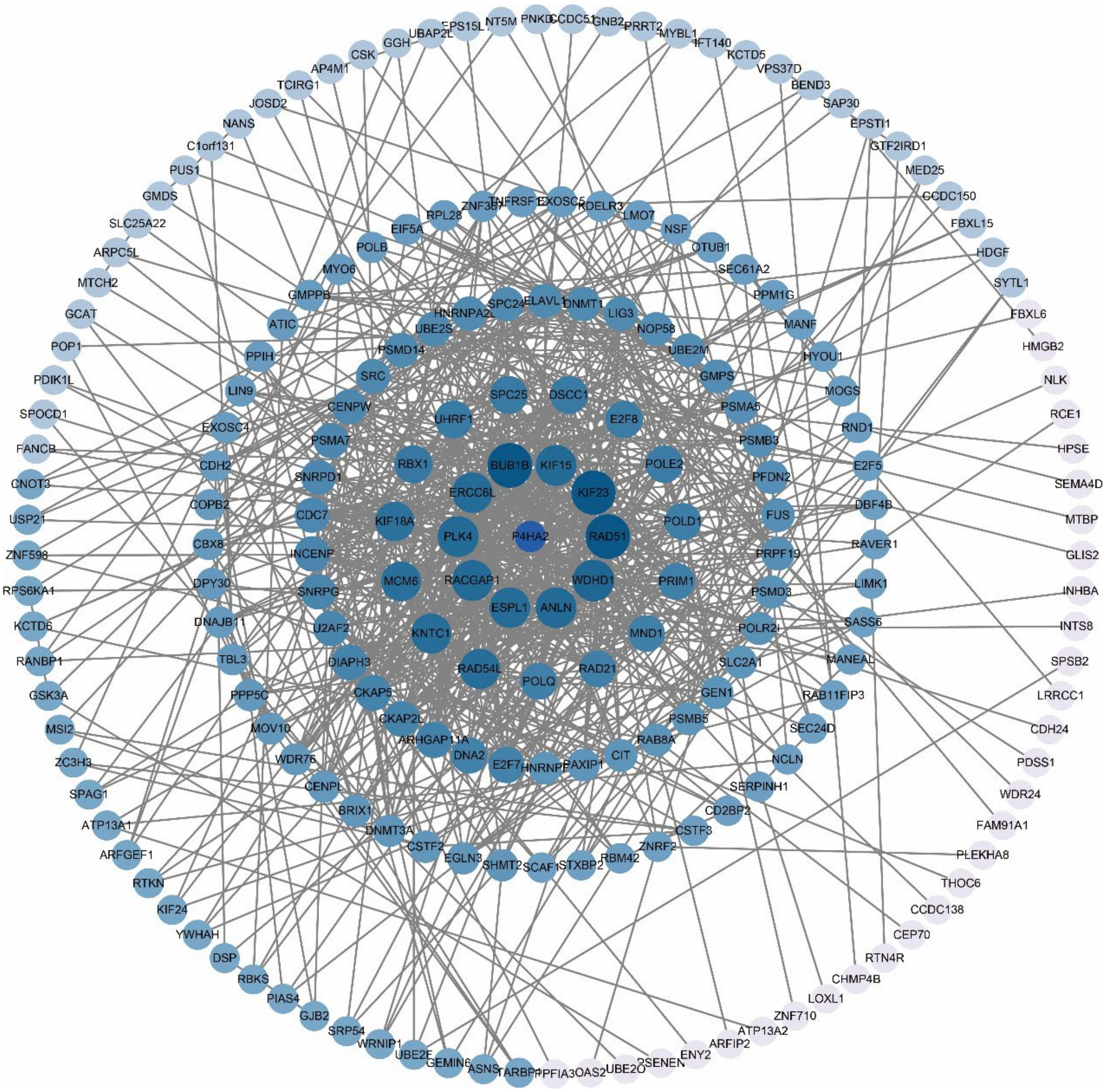

Figure S4. Interaction between P4HA2 and the radiation resistance gene panel.

Supplementary Tables:

Table S1. Differentially Expressed Genes in TCGA and GSE120798 and Antibody Information.

Table S2. The gene panel derived from Radiotherapy resistance.

Table S3. Mendelian randomization analysis of genes and their corresponding GWAS queue information related to the causal relationship between Radiotherapy resistance genomic panel and breast cancer.

Table S4. The marker gene of RRhighepi.

Table S5. Intersection of RRhighepi and Genes Filtered by Mendelian Randomization for Prognostic Model Construction (ML.genes).
